# Flavivirus and Filovirus EvoPrinters: New alignment tools for the comparative analysis of viral evolution

**DOI:** 10.1371/journal.pntd.0005673

**Published:** 2017-06-16

**Authors:** Thomas Brody, Amarendra S. Yavatkar, Dong Sun Park, Alexander Kuzin, Jermaine Ross, Ward F. Odenwald

**Affiliations:** 1 Neural Cell-Fate Determinants Section, NINDS, NIH, Bethesda, Maryland, United States of America; 2 Division of Intramural Research Information Technology Program, NINDS, NIH, Bethesda, Maryland, United States of America; Oxford University Clinical Research Unit, VIET NAM

## Abstract

**Background:**

Flavivirus and Filovirus infections are serious epidemic threats to human populations. Multi-genome comparative analysis of these evolving pathogens affords a view of their essential, conserved sequence elements as well as progressive evolutionary changes. While phylogenetic analysis has yielded important insights, the growing number of available genomic sequences makes comparisons between hundreds of viral strains challenging. We report here a new approach for the comparative analysis of these hemorrhagic fever viruses that can superimpose an unlimited number of one-on-one alignments to identify important features within genomes of interest.

**Methodology/Principal finding:**

We have adapted EvoPrinter alignment algorithms for the rapid comparative analysis of Flavivirus or Filovirus sequences including Zika and Ebola strains. The user can input a full genome or partial viral sequence and then view either individual comparisons or generate color-coded readouts that superimpose hundreds of one-on-one alignments to identify unique or shared identity SNPs that reveal ancestral relationships between strains. The user can also opt to select a database genome in order to access a library of pre-aligned genomes of either 1,094 Flaviviruses or 460 Filoviruses for rapid comparative analysis with all database entries or a select subset. Using EvoPrinter search and alignment programs, we show the following: 1) superimposing alignment data from many related strains identifies lineage identity SNPs, which enable the assessment of sublineage complexity within viral outbreaks; 2) whole-genome SNP profile screens uncover novel Dengue2 and Zika recombinant strains and their parental lineages; 3) differential SNP profiling identifies host cell A-to-I hyper-editing within Ebola and Marburg viruses, and 4) hundreds of superimposed one-on-one Ebola genome alignments highlight ultra-conserved regulatory sequences, invariant amino acid codons and evolutionarily variable protein-encoding domains within a single genome.

**Conclusions/Significance:**

EvoPrinter allows for the assessment of lineage complexity within Flavivirus or Filovirus outbreaks, identification of recombinant strains, highlights sequences that have undergone host cell A-to-I editing, and identifies unique input and database SNPs within highly conserved sequences. EvoPrinter’s ability to superimpose alignment data from hundreds of strains onto a single genome has allowed us to identify unique Zika virus sublineages that are currently spreading in South, Central and North America, the Caribbean, and in China. This new set of integrated alignment programs should serve as a useful addition to existing tools for the comparative analysis of these viruses.

## Author summary

Flaviviruses, including Zika and Dengue viruses, and Filoviruses, including Ebola and Marburg viruses, are significant global public health threats. Genetic surveillance of viral isolates provides important insights into the origin of outbreaks, reveals lineage heterogeneity and diversification, and facilitates identification of novel recombinant strains and host cell modified viral genomes. We report the development of EvoPrinter, a web-accessed alignment tool for the rapid comparative analysis of viral genomes. EvoPrinter superimposes alignment data from multiple pairwise comparisons onto a single reference sequence of interest, to reveal both similarities and differences detected in hundreds of selected viral isolates. Evoprinter databases provide easy access to hundreds of non-redundant Flavivirus and Filovirus genomes. allowing the user to distinguish between sublineage identity SNPs and unique strain-specific SNPs, thus facilitating analysis of the history of viral diversification during an epidemic. EvoPrinter also proves useful in identifying recombinant strains and their parental lineages and detecting host-cell genomic editing. EvoPrinter should serve as a useful addition to existing tools for the comparative analysis of these viruses.

## Introduction

Flaviruses, including Dengue, Yellow Fever, Japanese Encephalitis and West Nile viruses, are significant public-health pathogens responsible for wide-spread epidemics. Recently, another member of this genus, Zika virus (ZIKV), has emerged as a global public health threat (reviewed in [1]. Two major ZIKV lineages have been recognized: an African lineage first detected in the Uganda Zika forest in 1947, and an Asian lineage, first isolated in South East Asia during the 1950s, that has since spread to the Americas (for review, [2, 3]). Phylogenetic analysis has revealed that both the African and Asian lineages can be further divided into distinct sublineages or groups [4, 5]. Recent studies have also shown that ongoing epidemics are accompanied by the continued diversification of viral sequences via accumulation of base substitutions and recombinant exchanges between related sub-groups [3, 6, 7]. Members of the Flavivirus genus have been grouped based on their vectors (reviewed in [8]). Mosquito-borne human pathogens include ZIKV, Yellow Fever virus, four Dengue virus species, St. Louis and Japanese encephalitis viruses, and West Nile virus, along with other highly diverse less-characterized groups for review, [8]. Although mosquitos are considered the primary vector for ZIKV transmission, recent studies have identified human to human transmission via sexual contact [9].

Analysis of Filovirus human outbreaks during the last 49 years, from the initial 1967 Marburg virus outbreak in Germany through the most recent 2014–15 Ebola virus epidemic in West Africa and in the Congo, indicates that these pathogens will continue to pose serious public health risks (reviewed in [10–12]. Ebola virus species involved in these outbreaks and other non-human infections include the Zaire, Sudan, Taï Forest, Reston and Bundibugyo species, with the Zaire strains responsible for the most extensive human outbreak [13, 14]. Likewise, multiple Marburg outbreaks have occurred in Kenya, the Congo, Angola, Uganda and South Africa (for review, [11, 15]. Studies indicate that each Filovirus genus may have its own particular transmission cycle that includes non-human primates, bats, rodents, domestic ruminants, mosquitoes and ticks (reviewed in [16]). While bats are considered the primary reservoir for many of these viruses [17, 18], studies on humans that survive acute Ebola/Zaire infections reveal the presence of persistent active virus within immune-privileged or tissue sanctuary sites [19].

Phylogenetic analyses of both Ebola and Marburg strains responsible for human and non-human primate hemorrhagic fevers reveal that genetically identifiable strains from distinct lineages are associated with individual outbreaks; during these outbreaks, evolving sublineages have emerged [20–27]. For example, sequence analysis of Ebola isolates collected during the 2014–2015 West African Zaire/Makona outbreak has revealed the presence of multiple distinct sublineages that can be temporally traced to an initial Guinea strain that diversified during its spread into Liberia and Sierra Leone [13, 28–31].

The availability of hundreds of Flavivirus and Filovirus genomic sequences is an important resource for acquiring insights into the evolution of these pathogens [32, 33]. Using current web-accessed alignment tools, when multiple viral genomes are compared, alignments are often difficult to visually assimilate given the large size of their readouts. For example, a ClustalW alignment [34] of 14 ZIKV strains produces a 51-page readout. In addition, web-accessed alignment programs restrict the number of viral isolates that can be compared in an individual alignment. To circumvent these limitations, we have developed a multi-genome alignment method that can superimpose hundreds of one-on-one alignments to reveal sequence polymorphisms and conservation as they exist within a sequence of interest [35, 36]. Individual one-on-one input:database alignments can also be accessed directly from the input-centric readouts.

The combined EvoPrinter/Clustal alignment algorithms described here access databases of hundreds of Flavivirus or Filovirus genomes, allowing the user to input a full or partial viral sequence to initiate a comparative analysis. EvoPrint readouts identify sequences shared by all selected strains, in addition to highlighting (through color-coding) unique base substitutions and those shared by subsets of database entries. EvoPrinter databases currently contains 1,094 Flavivirus entries including 148 ZIKV strains and 460 Filovirus genomes with 393 Zaire isolates from the recent West African Ebola outbreak. To demonstrate the utility of these comparative tools, we show how 1) alignment readouts highlight unique bases in both the input and database sequences; 2) multiple sublineages are identified within ongoing Florida, Dominican Republic, Puerto Rico, and Brazil ZIKV outbreaks; 3) SNP analysis of other ZIKV strains also reveals different Central American, Caribbean and Chinese sublineages; 4) novel Dengue2 and Zika recombinant viruses and their parental lineages were identified using differential SNP pattern screens; 5) SNP patterns differentiate between Ebola/Zaire sublineages; 6) host cell A-to-I hyper-editing within Ebola and Marburg genomes is identified by SNP profiling and 7) inter-species multi-genome Ebola virus alignments can identify ultra-conserved sequences.

## Materials and methods

Flavivirus and Filovirus EvoPrinter search and alignment algorithms allow for rapid one-on-one or multi-genome comparisons of either a user supplied viral sequence or a database sequence selected from hundreds of database genomes. By superimposing sequence homology data from either a single or an unlimited number of one-on-one alignments onto a selected reference sequence, EvoPrinter readouts provide an uninterrupted view of polymorphisms as they appear within the genomes of interest and allow direct access to individual alignments by expanding readout sequence lines. The following is a description of the EvoPrinter databases, alignment algorithms and readouts. The EvoPrinter programs and tutorials are found at: https://evoprinter.ninds.nih.gov/evoprintprogramHD/evphd.html.

### EvoPrinter databases

Genomic sequences were curated from the NCBI/Genbank database [32], and additional information about virus strains was obtained from the Virus Pathogen Database [33]. To ensure that duplicate genomes do not interfere in the identification of uniquely shared sequences among different strains, redundant entries (detected by BLAST or Evoprinter alignments) were excluded. Database genome names contain the following information: species, NCBI designation, country of origin and year of isolation. When available, additional information is included in the names, such as lineage assignments, group designations and/or serotypes [14, 25, 37–44]. A lineage represents a set of genomes that differ from others within a species by a unique assemblage of sequence polymorphisms when compared to other species members. Different lineages are often marked by greater than 50 unique lineage-specific base differences.

In addition to FASTA formatted sequences, each entry was formatted for enhanced-BLAT (eBLAT) alignments to speed initial database searches [36, 45]. For eBLAT alignments, each genome was indexed into non-overlapping 11-mers, 9-mers and 6-mers and used to generate independent BLAT alignments that are superimposed to produce an eBLAT readout [36]. As of April 2017, the Flavivirus EvoPrinter database contains 1,094 non-redundant genomes that include the following: 574 Dengue (groups 1–4); 37 St. Louis Encephalitis; 115 West Nile; 110 Japanese Encephalitis; 70 Yellow Fever; 148 Zika; 8 Aroa-related; 7 Edgehill-related; 3 Entebbe-related; 3 Natya-related; 2 Spondweni-related; 12 Yaounde-related; 14 Insect-specific; 4 No Known Vector; 5 Seabird Tick-associated; and 8 Tick-borne genomes. Flavivirus groupings correspond to those previously described [8, 46, 47]. Databases will be updated when new genomes are submitted to NCBI.

The Filovirus database currently consists of 460 genomes that include 66 Marburg strains and 393 Ebola (371 Zaire, 10 Sudan, 7 Reston, 4 Bundybuygo, and 1 Taï Forest) isolates. Also included in the database is a single Cuevavirus genus strain, Lloviu Cuevavirus, isolated from European cave bats [48, 49].

### EvoPrinter alignment steps, computational processing and readouts

To initiate the comparative analysis of a user-provided sequence, an eBLAT search is performed to identify database genomes that closely match the input sequence [36]. User-supplied sequences can range from 100 bases to complete genomes. Once the eBLAT search identifies the input species, one-on-one Clustal alignments using the alignment algorithms developed by [34] are generated between the input sequence and the intra-species database genomes. Although BLAT alignments are significantly faster than Clustal comparisons, aligning bases at or near sequence ends are often missed due to insufficient K-mer alignment target lengths. Pairwise alignments are then converted to distinguish between aligning bases (upper case) and non-aligning bases (lower case) within the input sequence for each comparison [45]. This input-centric format allows for the superimposition of alignment data from an unlimited number of pairwise comparisons [35, 36]. In addition, holding one-on-one alignment data in memory instead of multi-genome alignments allows for user-customized comparisons.

To achieve higher throughput volumes and processing speeds, we wrote a Java-based program that employs multithread parallel processing [50] to generate pairwise alignments concurrently. By random allocation of 144 computational threads, database search and alignment processing speeds are significantly enhanced using a Hewlett Packard 2.5GHz/512 GB RAM; 4 socket, 18-core processor server operating with the RedHat Enterprise Linux 6 operating system. User-provided Flavivirus sequences (including full genomes) are automatically aligned to all intra-species database genomes and, to speed up processing times, alignments to the larger 18 kb Filovirus genomes are done incrementally, with the initial alignment round to the top ten eBLAT scoring Filovirus genomes. Additional database genomes can then be added to include strains of interest.

From the genome selection tree, the user can select genomes for single or multi-genome comparisons with the input sequence. The genome selection page orders the one-on-one alignments, based on the number of base mismatches with the input sequence (least to most). The selection page allows the user to 1) view individual alignments with the input sequence by selecting the genome of interest; 2) view multi-genome superimposed alignments of all or a selected subset of genomes in order to either identify shared or conserved sequences via an EvoPrint readout or to highlight sequence differences by generating an EvoDifference print readout, and 3) initiate inter-species alignments. By moving back and forth between the genome selection page and alignment readouts, the user can quickly add or remove viral strains from the comparative analysis.

**Fig 1:**
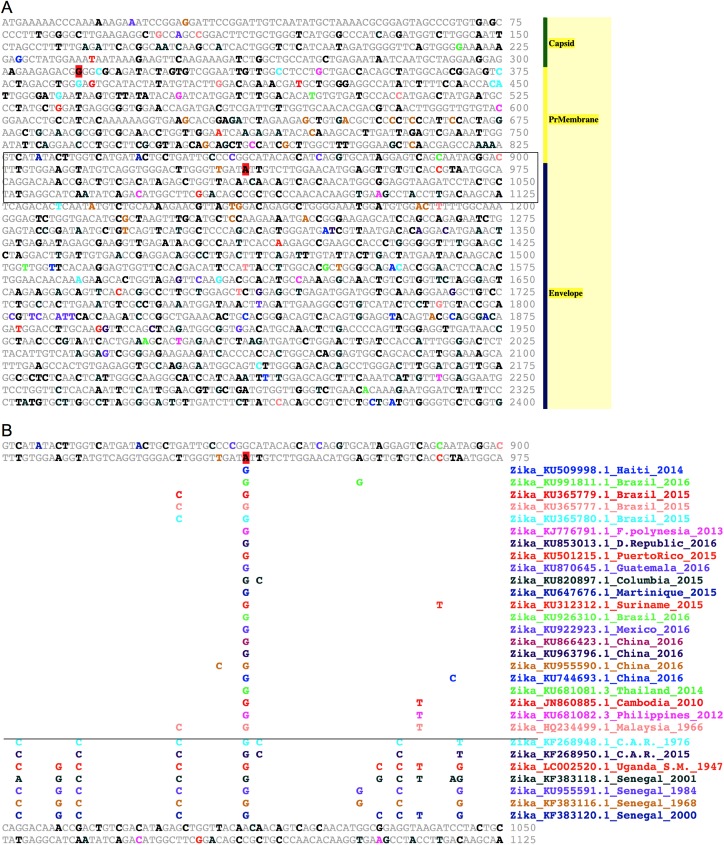
Zika virus EvoDifference prints highlight conserved bases and sequence polymorphisms within Asian and African lineages. (**A**) An EvoDifference printout of the first 2,400 bases of the Zika_KU321639.1_Brazil_2015 polyprotein ORF that spans the Capsid, Pre-Membrane and Envelope encoding regions was generated with 22 Asian/Western Hemisphere and seven African isolates (listed in panel B). Pair-wise alignments between the input sequence (KU321639.1_Brazil) and the database genomes are superimposed to identify: 1) bases identical in all examined genomes (gray); 2) bases that differed in only one of the genomes (colored coded to match the font color of that genome name listed in panel B); 3) bases that differ in two or more database genomes (black); and 4) bases that are unique to the input sequence (red highlighted, black). Line numbers indicate the last base of each line. Seventy-five bases per line were selected to vertically stack codons to highlight the frequent codon wobble position differences for essential amino acids. The boxed sequence (bases 826 to 1,125) is shown in panel B. (**B**) To reveal alignment details, sequence line number 975 was expanded by clicking on the number. Database genomes are ordered by their total number of base differences from the input sequence (least to greatest). Base differences are shown for each pair-wise alignment. Note that the more evolutionary divergent African isolates (positioned below the horizontal line) have the highest number of SNP differences with the Brazilian reference sequence. Individual one-on-one alignments of the input reference sequence with database genomes can be accessed by double-clicking on the genome name of interest.

**Fig 2:**
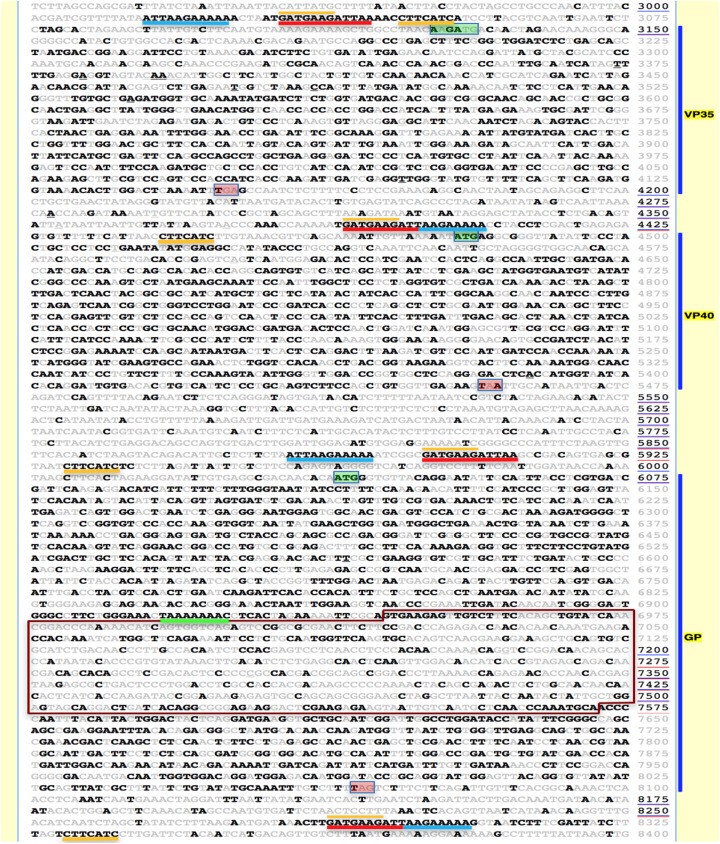
A multi-genome Ebola virus EvoPrint reveals conserved gene regulatory elements, essential amino acid codons and sequence variability within the glycoprotein surface domain ORF. The Zaire_lin6_Kissidougou_GIN_C15_KJ660346.2_2014 genome was EvoPrinted with 271 non-redundant Ebola virus genomes including 269 Zaire isolates and the TaïForest_lin1_Cote_dIvoire_FJ217162.1_1994 and Bundibugyo_lin1_Uga_FJ217161.1_2008 strains (EvoPrint database Zaire strains used to generate the print are available upon request). The EvoPrint highlights sequences within the input that are shared by all database genomes included in the analysis (bold black) and those bases that are different in one or more of the aligning genomes (gray). Shown are 5,475 bases of the full genome *EvoPrint*, starting in the 3’UTR of the NP gene and ending within the 5’UTR of VP30 and covering the VP35, VP40 and GP genes. Blue vertical bars indicate protein encoding ORFs. To highlight conserved codons and their variable wobble positions, the readout uses 75 bases per line. Conserved transcription start and stop sites are noted with blue and red underlining, respectively. The EvoPrint *also* identified a third conserved repeat element positioned 3’ of the transcription start signals (yellow underlined). Secondary structure predictions indicate that the sequence may form a stem-loop structure by base pairing to its reverse complement sequence within the transcription start signal (indicated by yellow over-lines) (reviewed in [47]). The conserved GP mRNA translational editing sequence is underlined green and its mucin-like domain coding sequence is boxed in red. ORF translational start and stop codons are boxed green and red, respectively. While the initiation ATG methionine codon for the VP40 and GP genes are conserved in all genomes, expanding the readout line that contains the translation start for VP35 reveals that both the Bundibugyo and Taï Forest species differ from the Zaire strains by base substitutions that generate start codons flanking the 5’ end of the Zaire ORF start (the positions of both are indicated by the elongated green box). Expanding sequence lines that contain termination codons reveals that while their positions are conserved, the three species use different stop codon combinations: for VP35, Zaire strains have TGA, Bundibugyo has TAA, and Taï Forest has TAG; for VP40, Zaire strains have TAA, while both Bundibugyo and Taï Forest have TGA; for the GP gene, Zaire strains have TAG; Bundibugyo has TAA; and Taï Forest has TGA. Underlined sequence line numbers indicate that sequence gaps were inserted in one or more of the genomes to optimize alignments: these can be viewed by clicking on the line number and then selecting the underlined genomes to view one-on-one alignments with the input sequence.

Sequence differences in multi-genome EvoDifference print readouts are color-coded to highlight base differences that are 1) unique to the input, 2) differ in only one of the database genomes, or 3) differ in two or more of the database entries (Fig 1). While sequence identity among the aligning genomes is indicated by gray-colored text in the EvoDifference print, conserved sequences within an EvoPrint readout are denoted by black text (Fig 2) and less conserved sequences are shown in gray font highlighted in green. In addition, bases that are unique to the input sequence and not present in any of the database genomes included in the EvoPrint readout are highlighted in red. The start and stop translation codons of open reading frames are highlighted when included in the alignment. For Flaviviruses, protein boundaries for the processed polyprotein are annotated (positions taken from the Virus Pathogen Resource [51]). Sidebars to the right of the readouts delineate protein encoding ORFs.

Sequence lines in both EvoDifference and EvoPrint readouts can be expanded to view the alignment details for each of the database genomes and, by selecting a virus strain listed in the readout, the user can view its one-on-one alignment with the input sequence. Amino acid alignments can also be viewed from one-on-one ORF alignments, to allow the user to assess whether nucleotide changes result in different encoded amino acids. A tutorial that details these alignment steps is available at the Flavivirus or Filovirus EvoPrinter websites via the EvoPrinter homepage (https://evoprinter.ninds.nih.gov/evoprintprogramHD/evphd.html).

### Pre-aligned libraries

As an alternative to a user-provided sequence analysis, a database genome can be selected as the input reference sequence for either individual or multi-genome alignments. EvoPrinter keeps a library of one-on-one alignment data between all Flavivirus database entries and a separate library for Filovirus database alignments that can be accessed for rapid comparative analysis. As with the user supplied input sequence search, database alignments are ordered on the genome selection page based on numbers of base mismatches compared to the input and individual alignments can be viewed by clicking on the database genomes.

### Identification of lineage or sublineage identity SNPs

**Fig 3:**
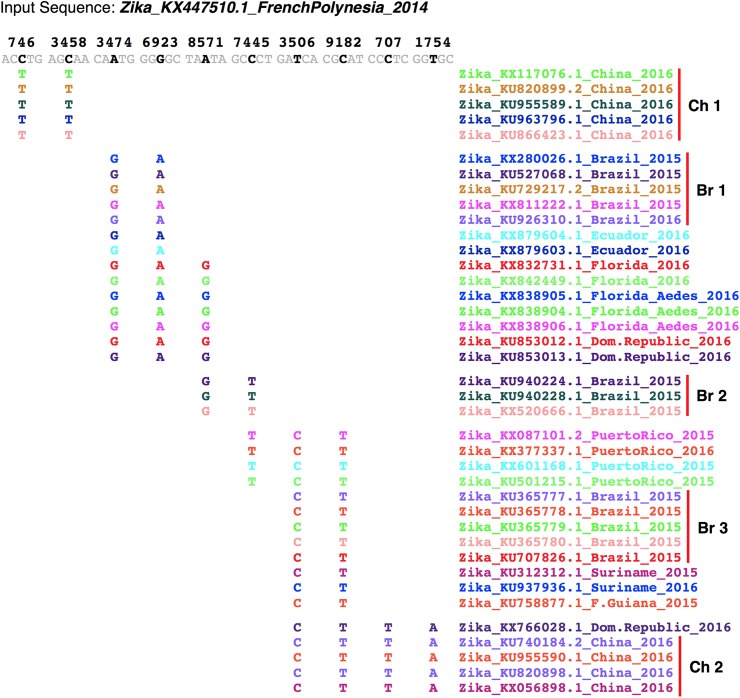
SNP patterns resolve different China and Brazil ZIKV sublineages that share identity SNPs with isolates from different countries. Shown are 10 identity SNPs within the Zika_KX447510.1_FrenchPolynesia_2014 genome that were identified from an EvoDifference print of 39 strains from China, Brazil, Ecuador, Florida, Dominican Republic, Puerto Rico, Suriname and French Guiana. The genomic positions of the identity SNPs are indicated above the French Polynesian reference sequences (horizontal sequence line). Gray-colored bases indicate that all genomes agree with the reference sequence and only database genome sequences that differ from the input sequence are shown and are color-coded to match the font color of the database genome name. Vertical bars highlight different China (Ch1 and Ch2) and Brazil (Br1-3) sublineages. Database genomes are grouped according to their shared SNPs. Note, the KX66028_Dominican Republic_2016 strain differs from members of the Chinese Ch2 sublineage by only 14 to 17 bases.

**Fig 4:**
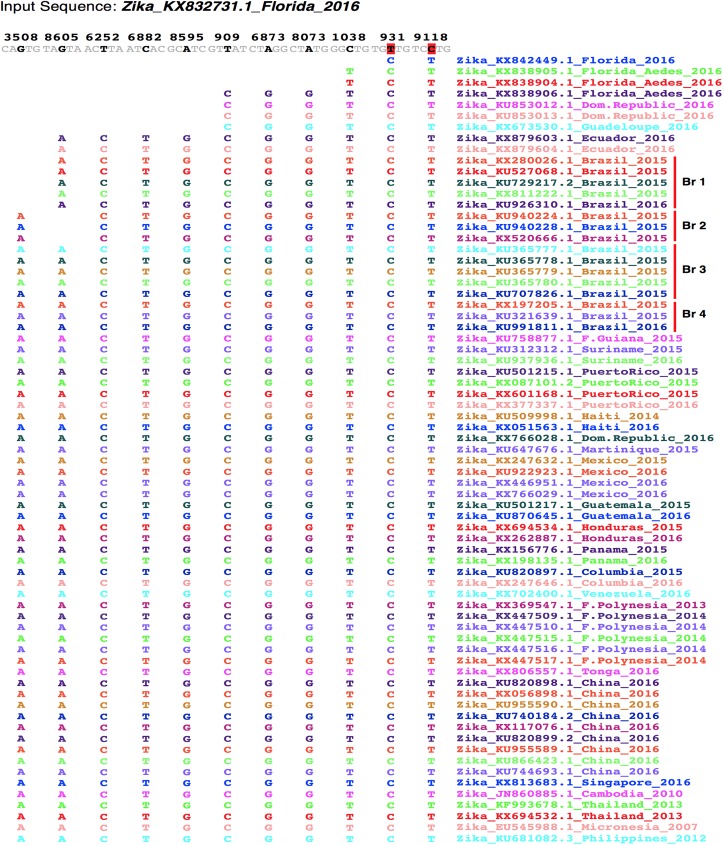
ZIKV evolutionary divergence inferred from shared base substitutions at multiple ancestral nucleotide positions. An EvoDifference print of the Zika_KX832731.1_Florida_2016 strain with 71 ZIKV strains from 24 different countries identifies nucleotide positions that differ only in a subset of isolates, while the other strains included in the analysis have maintained an ancestral base at these positions. Shown are 11 of 19 ancestral base positions that have undergone identical substitutions in subsets of genomes. For example, at position 3508 in the reference genome, there was an A->G substitution within all members of the Brazil Br1 sublineage and isolates from Ecuador, Guadeloupe, Dominican Republic and Florida. The readout also revealed other positions with fewer and fewer strains with the same identity SNP and base changes that are unique to the KX832731.1_Florida_2016 strain that are not found in other Florida strains nor in any of the other Asian lineage strains. Genomic position designations within the input reference sequences are shown above the horizontal KX832731.1_Florida_2016 sequences. Strains are grouped according their countries of isolation and the different Brazil sublineages (Br1-4) are grouped with vertical bars. Only database sequences that differ from the reference sequence are shown.

To resolve different lineages and/or sublineages, the user should select ten or more genomes that have similar mismatch numbers with the input reference sequence and generate a multi-genome EvoDifference print. On the genome selection page, the bracketed numbers after the database name represent the number of base mismatches with the input sequence. In the readout, bases in black text indicate two or more database mismatches, and when these multi-genome differences are identical in two or more strains they frequently represent lineage or sublineage identity SNPs. In other words, when multiple strains have the same base substitutions these SNPs can be considered markers of lineage progression. By expanding readout lines that contain multiple mismatches, sublineages can be differentiated by their uniquely shared differences with the input (see Figs 3 and 4).

### Detecting recombinant Flaviviruses

One-on-one EvoDifference print SNP patterns can be used to identify recombinant viruses and their parental lineages. Virus strains that are closely related to the input sequence, as revealed by low mismatch numbers (listed after the database genome name on the Genome Selection Tree), usually have randomly distributed base differences throughout their pairwise alignments with the input sequence. Discontinuity in mismatch scores between related database genomes, as seen by a sudden jump in score values, are often due to one of two reasons. First, a higher score can indicate a sublineage difference and in this case, the increased SNPs are randomly distributed throughout the alignment. Second, the higher score could indicate a recombinant exchange, and in this case, a cluster of high-density SNPs (a recombinant fragment from a more divergent minor parent) would be flanked by regions of lower SNP densities (from the major parent). Alternatively, if the recombinant is aligned with a member of the minor parental lineage, a significantly reduced low-SNP density region (corresponding to the above high SNP density cluster) is flanked by regions of higher SNP densities (from the major parent).

**Fig 5:**
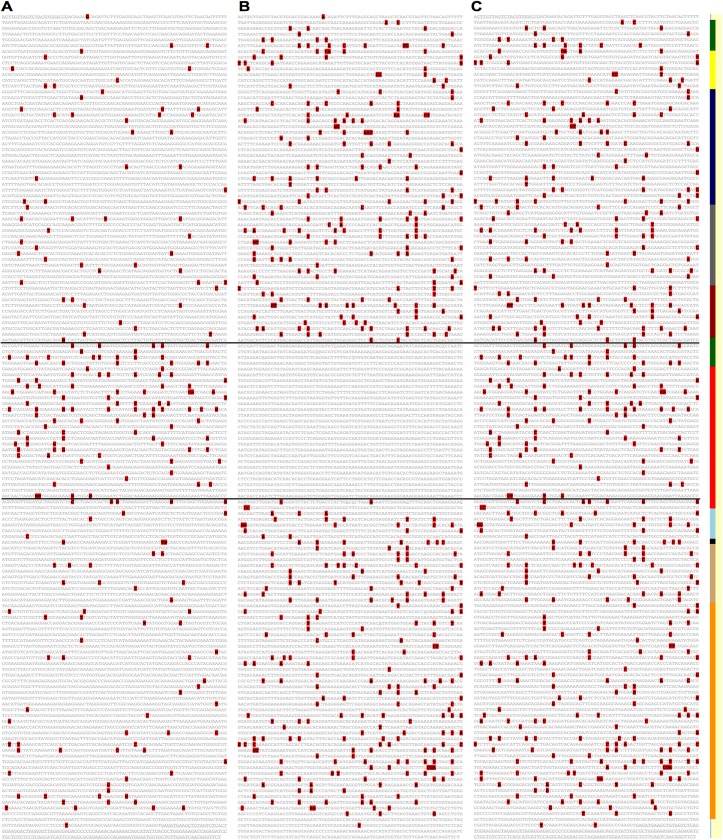
EvoDifference prints identify a recombinant exchange between New Guinea and Puerto Rico Dengue2 viral strains. Differential SNP patterns reveal that the Dengue2_GQ398269.1_PuertoRico_1994 isolate is a recombinant made up of genomic fragments from different parental sublineages. Starting with their 5’ ends, each alignment covers 10,724 bases. Gray-colored bases indicate sequence identity and red highlighted sequences identify base differences. The input reference sequence is listed first followed by aligning database genomes. (**A**) Alignment of Dengue2_KF955363_PuertoRico_1986 (major parental lineage) and Dengue2_PuertoRico GQ398269.1_1994 (recombinant); (**B**) Dengue2_AF038403.1_NewGuinea_1988 (minor parental lineage) and GQ398269.1_PuertoRico (recombinant); (**C**) Pairwise alignment of the major and minor parental lineage members KF955363_PuertoRico and AF038403.1_NewGuinea isolates, respectfully. Horizontal lines serve as approximate guides for recombination boundaries. Flanking vertical color bars indicate the ORF encoding positions of the poly-protein.

**Fig 6:**
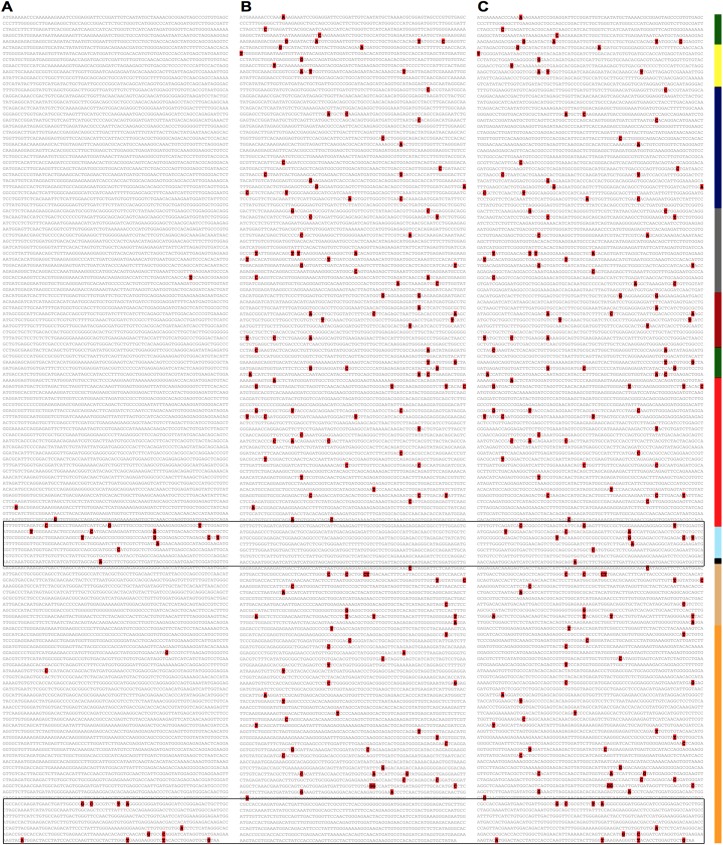
Identification of a novel Chinese/Cambodian Zika recombinant virus via EvoDifference print SNP patterns. Pairwise EvoDifference prints of the Zika_KU866423.1_China_2016 genome with other Asian strains identify SNP patterns that differ in Cambodian and Chinese sublineages. Gray-colored bases indicate sequence identity and red highlighted sequences identify base differences. Each alignment covers 10,272 bases representing the complete poly-protein ORF. Horizontal lines delineate regions of recombinant exchange (bases 6,315 to 6,783 and bases 9,777 to 10,272). Note that the recombinant fragment boundaries are estimates and may extend into flanking regions that are identical in both the recombinant and parental genomes. Reference sequence listed first followed by database genome: (**A**) Zika_KU963796.1_China_2016 (major parental sublineage) aligned to Zika_KU866423.1_China_2016 (recombinant); (**B**) Zika_JN860855.1_Cambodia_2010 (minor parental sub-group) aligned to KU866423.1_China recombinant; (**C**) Alignment of the major sublineage (KU963796.1_China) with the minor parental sublineage (JN860855.1_Cambodia). Flanking vertical color bar indicates approximate positions of the encoded viral proteins as in Fig 5.

To identify members of the minor parental lineage, the database search is repeated using the region of the input sequence that generates the high SNP density cluster along with flanking sequences of the putative recombinant strain. If members of the minor parental lineage are present in the database, they will likely have the lowest mismatch numbers when compared to the other database genomes. By repeating the initial search using the complete or nearly complete recombinant genome and then comparing one-on-one alignments with members of both parental lineages, the genomic region that generates the high SNP density when aligned to a major parental lineage strain (Figs 5A and 6A) will show near identity within the corresponding region when aligned to a minor parental strain (Figs 5B and 6B). If a member of the minor parental strain is detected first, the members of the major parental lineage can be identified in database searches by using the low SNP density region plus its flanking higher SNP density regions and examining high mismatch scoring strains.

Differential SNP patterning can also be used to identify recombinant strains that are decedents of multiple rounds of recombinant exchanges with different partners. For example, if not all of the high SNP density clusters observed in the recombinant / major parental lineage alignment have corresponding “SNP clearings” when aligned to a member of the minor parental lineage, then the recombinant strain most likely is a mosaic of different recombination events involving multiple partners. To confirm putative recombinants, we recommend that additional recombinant detection programs be employed such as the Recombination Detection Program [52].

### Identifying host cell A-to-I editing of Filovirus genomes

Both one-on-one and multi-genome EvoDifference prints of related Ebola or Marburg strains can be used to identify genomic sequences that have undergone A-to-I editing by host cell adenosine deaminases. When the conversion occurs within the replicative template of Filoviruses, the inosines are read as guanine residues resulting in T/U -> C substitutions in the negative stranded RNA genome (for review, [53]). In both one-on-one and multi-genome EvoDifference prints, hyper-editing appears as clusters of T or C unique substitutions depending on whether the editing occurred in the input sequence or database genome.

## Results and discussion

We have modified the EvoPrinter phylogenetic footprinting tool for the rapid comparative analysis of Flavivirus and Filovirus genomic sequences. Its alignment algorithms superimpose alignment data from an individual or up to hundreds of pairwise alignments, highlighting both sequence conservation and base differences within the user’s input sequence and database genomes. SNP pattern differences and conserved sequences can be viewed from readouts that highlight sequence differences (an EvoDifference print) or sequence conservation (an EvoPrint) (Figs 1 and 2). By expanding multi-genome readout lines, individual database alignments reveal SNPs that define lineages or sublineages, clusters of A-to-I host cell hyper-editing, and conserved sequence elements shared by all or a subset of database genomes. Differentially shared SNP patterns, identified in one-on-one EvoDifference print comparisons, also allow for the identification of recombinant viruses and their parental lineages.

### Characterizing Flavivirus lineage differences via SNP patterning

Resolving sublineages during a viral outbreak or epidemic facilitates the identification of the genetic heterogeneity among viral isolates, identifies the spread of related strains to different countries, and allows for the detection of recombinant variants. Based on phylogenetic analysis, previous studies have identified major ZIKV groups: two African groups, consisting of West and East African sublineages [3] and a diverse Asian/Western hemisphere lineage (for review, [54]). The West African group contains isolates primarily from Senegal and Cote-d’Ivoire, while the East African sublineages can be further resolved into isolates from Uganda and the Central African Republic.

EvoDifference print readouts can be used to highlight sequence differences among related and evolutionary distant ZIKV strains (Fig 1). Using the capsid, pre-membrane and envelope encoding region from the Zika_KU321639.1_Brazil_2015 strain as the reference input sequence, one-on-one alignments with twenty-nine ZIKV database genomes were selected to identify 1) bases that are unique to the input, 2) bases that differed in only one of the database genomes, 3) sequences that differed in two or more database genomes, and 4) sequences shared by the input and all selected database genomes (Fig 1A). Alignment details and the color-coded names of the database isolates included in the comparative analysis can be viewed by selecting line numbers (Fig 1B). In this example, sequence line number 975 was expanded to highlight SNPs that are unique to the input or database genomes and shared SNPs. The expanded sequence line also highlights the greater SNP density of the more divergent African isolates (located below the horizontal line) when compared to the Asian isolates (above the line). Database genomes are ordered by their total number of base differences when aligned to the input sequence (least to most). The genome ranking and base differences are also part of the database selection page.

Differentially shared SNP patterns among multiple ZIKV isolates can be used to resolve individual sublineages. For example, when 525 bases of the Zika_KF383118.1_Senegal_2001 NS5 coding region are used to generate an EvoDifference print with database genomes from different African sublineages, their base differences with the input Senegal isolate or SNP profiles resolve different sub-groups (S1 Fig), that correspond to previously described sublineages [3, 4, 55–57].

### EvoPrints highlight both conserved and less-conserved sequences

Phylogenetic footprinting, identifying evolutionary conserved sequence elements using multi-genome alignment protocols, has become an important tool for resolving essential genomic information [35, 58, 59]. A significant advantage of EvoPrinter is the ability to rapidly change the cumulative evolutionary divergence stringency of a multi-genome comparison. By moving between the genome selection page and the EvoPrint readout, one can quickly add or remove viral strains from the analysis to reveal different levels of conservation of essential elements, as they exist within genomes of interest. For example, to identify previous characterized Ebola virus conserved transcriptional start and stop regulatory elements (for review [60, 61]), we generated a multi-genome *EvoPrint* of the Zaire_lin6_Kissidougou_GIN_C15_KJ660346.2_2014 strain that included 271 non-redundant genomes from 3 Ebola species (269 Zaire, 1 Bundibugyo and 1 Taï Forest) (Fig 2). In addition to resolving transcriptional regulatory elements that flank each of the seven Ebola virus genes, the divergence stringency of the EvoPrint is sufficient to highlight essential amino acid codons by revealing their less-conserved wobble positions and identify the transcription editing site within the GP gene (Fig 2). The EvoPrint also delineates less-conserved intergenic regions and the evolutionarily variable GP mucin-like domain encoding region [62] (Fig 2).

As with the Filoviruses, near-base resolution of essential information is obtained with Flaviviruses. A multi-genome EvoPrint was generated using the YellowFever_GQ379162.1_Peru_2007 NS3 encoding region as the input reference sequence, comparing it with 15 South American and African Yellow Fever strains selected from the Yellow Fever database (S2 Fig). Together the 15 strains provide a cumulative evolutionary divergence sufficient to resolve essential bases, as evident from the less conserved codon wobble positions (S2A Fig). Flavivirus SNP differences can also be accessed by expanding readout lines of multi-genome EvoPrints. The shared SNP profiles of different Yellow Fever Virus sub-groups (S2B Fig). correspond to previously identified phylogenetic tree groupings [63].

### SNP profiling identifies sublineages within Zika virus outbreaks

Shared SNPs that highlight differences between groups of viruses serve as ancestry informative markers for identifying sublineages (for review, [64]). We call these identity SNPs (ID-SNPs), since they represent lineage markers for descendants of an earlier parental strain and multiple shared ID-SNPs, or profiles can be used to resolve different sublineages and illuminate ancestral relationships among ZIKV strains during spreading epidemics. Most ID-SNPs highlight differences between a sublineage and all other strains outside of the sublineage that have maintained the same ancestral base at those nucleotide positions (Figs 3 and 4).

Phylogenetic tree comparisons of Asian/Oceania strains have revealed that the South American epidemic (first identified in Brazil) derives from a distinct sublineage that arose from an outbreak in French Polynesia in 2013 [4, 55–57] (for review [4, 65, 66]). Our SNP profiles of Brazilian isolates reveal that they can be further divided into at least four different subgroups based on non-overlapping ID-SNP patterns shared among 20 isolates (Fig 3 and S3 Fig). For example, when the Zika_KX447510.1_FrenchPolynesia_2014 strain is used as the input reference genome and aligned to 13 Brazilian isolates, 3 subgroups (Br1-3) (each represented by multiple isolates) were distinguished by 22 ID-SNPs that are positioned throughout the genome (Fig 3). When isolates from China, Ecuador, Florida, Dominican Republic, Puerto Rico, Suriname and French Guiana are included in the analysis, all five of the Florida isolates, all of the Ecuador, and two of three Dominican Republic strains share ID-SNPs with the first Brazilian subgroup (Br1) but not with the Br3 subgroup (Fig 3). The second Brazil sublineage (Br2) shares ID-SNPs with Florida isolates and with the Puerto Rico strains but not with Br1 or Br3 (Fig 3). The alignment also reveals that the Puerto Rico, Suriname, French Guiana and a single Dominican Republic isolate share ID-SNPs with the third Br3 Brazil subgroup but not with the Br1 sublineage. In addition, while isolates from Florida and Puerto Rico represent two distinct subgroups, the ID-SNP patterns of isolates from the Dominican Republic reveal that one isolate is related to the Puerto Rico subgroup while the other two share ID-SNPs with the Florida subgroup (Fig 3). Interestingly, pairwise alignments between the Dominican Republic isolate that is related to the Puerto Rico subgroup, the Zika_KX766028.1_DominicanRepublic_2016 strain, and any of the China Ch2 sublineage members reveal near identity, suggesting that the Ch2 sublineage may have originated from the Caribbean (Fig 3 and S4 Fig). This possibility is further strengthened by the observation the China Ch2 strains share many ID-SNPs with isolates from Puerto Rico, Dominican Republic, Suriname, French Guinea, and members of the Brazil Br3 sublineage. In addition, these observations are in agreement with Zhang et. al., who report the presence of highly diversified ZIKVs that have been most likely imported into China [67].

Comparative analysis of isolates from the recent southern Florida outbreak identify ancestral ID-SNPs that together suggest a progressive evolutionary divergence away from other related strains and other members of the Asian lineage. For example, an EvoDifference print of the Zika_KX832731.1_Florida_2016 isolate with 71 other Asian/Oceanian/Western hemisphere strains (both related and evolutionarily distant) revealed ID-SNPs that are shared among Florida and Dominican Republic isolates while all other strains have maintained the same ancestral base at those positions (Fig 4). Our analysis also identified ancestral ID-SNPs that are restricted to just a subset Florida and Dominican Republic strains and ID-SNPs that only distinguish a subset of Florida isolates from all other Asian lineage strains. Taken together, the different subgroups indicate that progressive, multi-generation base substitutions at different genomic positions are playing a significant role in ZIKV divergence. In addition, the multi-genome analysis demonstrated that the KX832731_Florida strain has recently acquired three unique SNPs that are not shared by any of the other Asian/Oceanian/South American strains (two of the three unique SNPs are red highlighted in Fig 4).

We have also used ID-SNP profiles to search for additional Western Hemisphere sublineages by examining pair-wise alignments of South/Central American and Caribbean isolates. Our screen identified two Central American sublineages, differentiated from the Brazil Br1-4 subgroups by combinations of 15 ID-SNPs (S3 Fig). These subgroups contained isolates from Mexico, Guatemala, Honduras, Panama and Columbia. Strains from Mexico fall into either the first or second central American group. Our comparative analysis also revealed that the single Martinique isolate, Zika_KU647676.1_Martinique_2015, most likely originated from a Mexican strain as it differs from the Zika_KU922960.1_Mexico_2016 isolate by only 4 bases.

To examine sublineage heterogeneity among Asian and Southeast Asian ZIKV strains, we searched for ID-SNPs that group isolates from different locations. As indicated above, our SNP pattern screen revealed two Chinese subgroups that are differentiated by 31 ID-SNPs (Fig 3 and S4 Fig). Using Zika_KU955589.1_China_2016 as the input reference genome, our multi-genome analysis revealed that the Chinese Ch2 subgroup shares many ID-SNPs with Western hemisphere isolates, while the first China subgroup (Ch1) constitutes a distinct (perhaps older) Asian sublineage (Fig 3 and S4 Fig). The French Polynesian strains share six ID-SNPs with the Ch1 subgroup and the Tonga strain shares eight ID-SNPs, suggesting that strains from Tonga and French Polynesia may be evolutionarily positioned between the Chinese Ch1 sublineage and Western hemisphere isolates (S4 Fig).

### Identifying recombinant Flaviviruses

Genomic diversity among Flaviviruses is driven in part by homologous recombination between related strains, with their recombinant exchanges occurring in both protein encoding and noncoding sequences [3, 7, 68, 69]. Alignment programs that scan for changes in sequence homology within multiple genomes and methods that examine differential phylogenetic clustering using genomic sub-regions have been used to identify recombinants and locate approximate recombinant fragment boundaries [70, 71]. Evoprinter screens can also identify recombinants and resolve the approximate boundaries of their recombining fragments within parental lineages.

By examining a previously characterized Dengue2 recombinant, we show how SNP profiling can be used to identify recombinant strains and their parental sublineages (S5 Fig). Phylogenetic tree clustering analysis of the Dengue2_AF100466.2_Venezuela_ 1990 (Mara4) strain with other Dengue2 genomes revealed that Mara4 is the recombinant progeny of two distinct Dengue2 sublineages [71]. Differential phylogenetic clustering analysis revealed that the first ~500 bases of Mara4 are nearly identical to Dengue2 strains from Thailand, while the remaining genome is related to American strains [71]. Side-by-side EvoDifference SNP profile comparisons of the Mara4 recombinant with members of the parental sublineages (from Thailand and Jamaica; S5A and S5B Fig, respectively) demonstrate that the 5’ recombinant fragment originated from the minor parental Thailand sub-group (boxed region in S5 Fig). Note that, by convention, the strain that produces the highest SNP density within the recombinant region when aligned to the recombinant strain is designated as the major parental lineage, while the minor parental sub-group shares identity or near identity with the recombinant within the boundaries of the recombinant fragment. In this example, the differing parental SNP pattern boundaries are located at positions 594 (major parent) and 600 (minor parent), indicating that the recombinant exchange most likely occurred between bases 595 and 599 (S5 Fig).

One advantage of the genome SNP profiling is that recombinants and their parental lineages can be identified by differental SNP patterning. For example, Fig 5 identifies a novel Dengue2 recombinant strain. Side-by-side comparisons of SNP profiles generated from one-on-one EvoDifference prints of the Dengue2_GQ398269.1_PuertoRico_1994 strain with another Puerto Rico strain (Dengue2_KF955363.1_PuertoRico_1986) and with a New Guinea isolate (Dengue2_AF038403.1_NewGuinea_1988)–Fig 5A and 5B, respectively—revealed that the Puerto Rico_GQ398269.1 strain is the resultant progeny of a recombinant exchange between a member of a Puerto Rican subgroup (major parental sublineage) and a New Guinea sub-group member (minor parental sublineage) (Fig 5). The abrupt SNP density pattern change within the recombinant Puerto Rico/New Guinea strain alignment delineates an ~2,100 base region (spanning the NS2B and NS3 protein encoding sequences) that is identical in both the recombinant and New Guinea genomes (Fig 5B). Note that the higher density SNP cluster in the Puerto Rico (major parent)–recombinant strain SNP profile alignment corresponds to the region of identity shared between the recombinant and the minor parental strain (Fig 5A and 5B).

### Recombinant Zika viruses

Using SNP profiling, we have sought evidence of recombination within Asian and African ZIKV lineages. Our initial screen of the China Ch-1 sublineage isolates revealed that many are nearly identical, however, the SNP profile generated when the Zika_KU963796.1_China_2016 strain was aligned to Zika_KU866423.1_China_2016 identified two genomic regions that have significantly higher SNP densities when compared to flanking sequences (Fig 6A). Further analysis that included other Asian strains revealed that when the KU866423.1 strain was aligned to a Cambodian isolate, Zika_JN860885.1_Cambodia_2010, their genomes are identical in the same two regions that displayed higher density SNP clustering in the above KU866423.1—KU963796.1 comparison, but differ significantly in sequences flanking these regions (Fig 6). The matching genomic positions that have converse high SNP density vs. sequence identity reveals that the KU866423.1 strain is the recombinant progeny of the two separate parental sublineages, one from China (major parent) and the other from Cambodia (minor parent). The one-on-one SNP pattern comparisons also revealed that the recombinant strain is the product of two genomic exchanges, with one occurring in sequences that code for NS3, NS4A and 2K proteins, while the other in-frame exchange occurred within the 3’ end of the NS5 coding region. Notably, when members of the parental lineages are aligned, their SNP profiles do not reveal any significant changes in SNP densities that would flag these as recombinant strains (Fig 6C).

**Fig 7:**
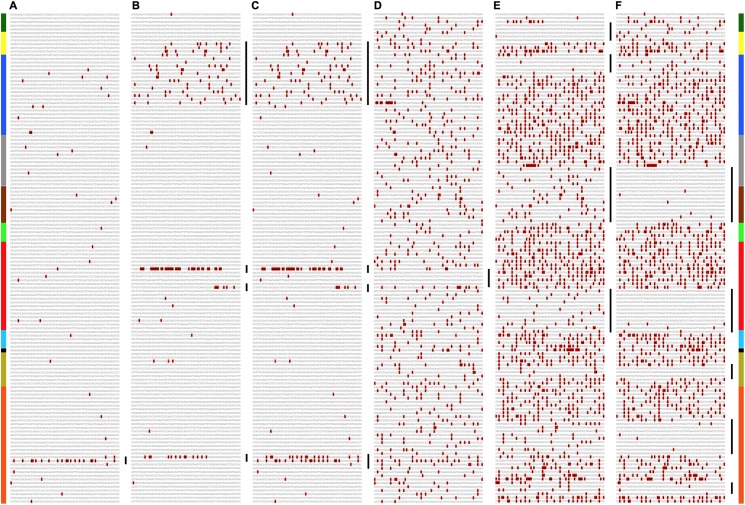
Putative recombination events within multiple African Zika virus strains identified by one-on-one EvoDifference prints. Shown are six pairwise polyprotein ORF alignments between four different African strains. Starting with the first codon, each alignment covers 9,975 bases (3,325 codons). Gray bases represent alignment identity and red highlighted bases identify sequence differences. The input reference sequence is listed first followed by the aligning database genome: (**A**) Zika_KF383119.1_Senegal_2001 aligned with Zika_LC002520.1_Uganda_S.M._1947; (**B**) Zika_KF383119.1_Senegal_2001 aligned with Zika_KF383118.1_Senegal_2001; (**C**) Zika_KF383118.1_Senegal_2001 aligned with Zika_LC002520.1_Uganda_S.M._1947; (**D**) Zika_KF383119.1_Senegal_2001 aligned with Zika_KF383116.1_Senegal, 1968; (**E**) Zika_KF383119.1_Senegal_2001 aligned with Zika_KF383120.1_Senegal_2000; (**F**) Zika_KF383116.1_Senegal_1968 aligned with Zika_KF383120.1_Senegal_2000. Vertical black bars to the right of each panel highlight regions with significant changes in SNP density indicating putative recombinant exchanges. Flanking vertical color bars indicate ORF positions of the encoded proteins (Capsid, green bases 1–366; Pre-Membrane, yellow 367–900; Envelope, dark blue 901–2400; NS1, gray 2401–3426; NS2A, brown 3427–4494; NS2B, green 4494–5885; NS3, red 5886–6345; NS4A, light blue 6345–6726; 2K, black 6727–6795; NS4B, tan 6796–7501; NS5, orange 7502–9975).

African lineage ZIKV recombinant strains have been described previously [3]. Consistent with these observations, EvoDifference prints of available African ZKIV strains have identified multiple one-on-one alignments that display significant changes in SNP densities within different regions of their polyprotein encoding sequences (Fig 7). For example, using the Zika_KF383119.1_Senegal_2001 as the input reference genome and examining other African strains, significant changes were identified in SNP densities within different genomic regions. Our initial multi-genome comparisons identified a 139-base region within the NS5 coding region that significantly differs from sequences within the original 1947 Uganda Zika forest sentinel monkey isolate and two other strains from Senegal and the Central African Republic (S6A Fig). Expanding the readout lines revealed that the Uganda and Senegal isolates are identical to adjacent but non-overlapping portions of the KF383119.1_Senegal reference sequence, while a Central African Republic strain shares many of the sequence differences of both the Uganda and Senegal isolates (S6B Fig).

Examination of other African strains also revealed SNP clustering within this region and other significant changes in SNP densities outside of the NS5 coding region. Similar to the China/Cambodia recombinant, many of the high-to-low-to-high SNP density changes indicate multiple recombination exchanges have occurred within these viruses (Fig 7). For example, alignment of the KF383119.1_Senegal with Zika_KF383118.1_Senegal strains identified three additional clusters of sequence differences; most notably, a putative recombinant fragment that spans the capsid and envelope encoding sequence (Fig 7B). Also note, the SNP cluster in panel A (that spans the NS5 encoding sequence) and the high density SNP cluster within the same genomic region shown in panel B were adjacent but non-overlapping (also highlighted in S6B Fig.pdf). High density SNP clusters were also identified in an EvoDifference print of KF383118.1_Senegal and the LC002520.1_Uganda (Fig 7C), with the NS5 SNP cluster expanded to include both NS5 high density SNP clusters (Fig 7A and 7B). The juxtaposition of high and low SNP densities within the one-on-one comparisons highlight putative recombinant exchanges, with one of the aligning strains most likely belonging to the major parental sublineage (Fig 7A–7C).

An EvoDifference print of the African KF383119.1 strain with an evolutionarily distant African strain, Zika_KF383116.1_Senegal_1968, shows extensive divergence throughout their coding sequences, with the exception of the centrally located NS3 encoding sequence (bases 5227 to 5556) (Fig 7D). In addition, pairwise alignments with other African strains uncovered evidence of additional African recombinant exchanges. For example, one-on-one SNP profiles of KF383119.1 or KF383116.1 strains with another highly divergent Senegal strain, Zika_KF383120.1_Senegal_2000 revealed multiple significant changes in SNP densities (Fig 7E and 7F, respectively). Although the KF383120.1 strain is considered to be inactive, given the presence of an internal in-frame stop codon [3], recent phylogenetic analysis reveals that the KF383120.1 strain belongs to a distinct African sublineage that includes other closely related functional strains [4].

### Filovirus Evoprinter

**Fig 8:**
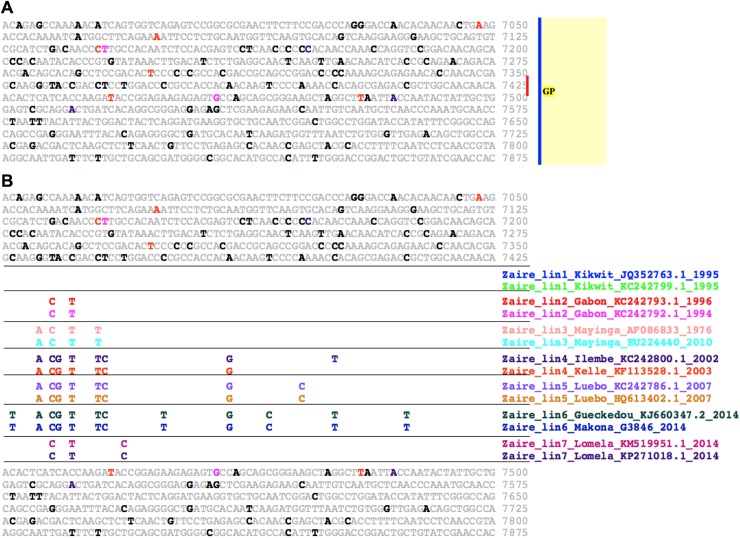
EvoDifference prints identify Ebola/Zaire virus lineage specific polymorphisms. Two representative strains from each Zaire lineage were chosen to illustrate lineage specific SNPs. (**A**) An EvoDifference print of reference sequence Zaire_lin1_Kikwit_AY354458.1_1995 bases 6975 to 7875 spanning the GP mucin-like domain was generated with pairs of each of the seven lineages. (**B**) Line 7475 expanded to reveal lineage-specific SNP patterns among the seven Zaire lineages. Note the first two lines are identical to the input reference sequence, while the other lineages are distinguished by their unique SNP patterns. Sequence color-coding as in Fig 1.

Filovirus database genomes are grouped according to their species and lineage designations [23, 25, 26, 37, 72]. A comparative analysis of the Zaire species identified seven lineages that make up three major groups: 1) the Kikwit (lin1), Gabon (lin2) and Mayinga (lin3) isolates taken together fall into a related group; 2) the Ilembe (lin4), Luebo (lin5) and Boende (lin7) together fall into a second group, and 3) the recent Zaire/Makona West African isolates (lin6) represent a more divergent lineage (Fig 8), in agreement with recent lineage designations [72]. Alignments of the other Ebola species revealed two Bundibugyo lineages, four Reston, six Sudan lineages and one Taï Forest (the sole sequence in this species). Consistent with previous studies, EvoDifference prints identified nine Marburg lineages [22, 73].

### Identifying Ebola/Zaire lineage and subgroup distinguishing SNP patterns

As an example of using SNP patterning to resolve different Filovirus lineages, we show how a multi-genome EvoDifference print of the Zaire_lin1_Kikwit_AY354458.1_1995 GP gene mucin-like domain encoding sequence [62] with other Zaire reference strains can identify different lineage-specific SNP patterns (Fig 8). By selecting a readout line number (line 7425 in this case), sequence differences are revealed among the different aligning lineage pairs, allowing an assessment of which bases conform to the input sequence and which are different and unique to a single lineage or shared by various other lineages (Fig 8B). Interestingly, most of the SNPs within the mucin domain are T/U->C substitutions and may be the result of host cell A-to-I RNA editing (discussed below).

Phylogenetic analyses coupled with retrospective epidemiological studies of the recent West African Ebola/Zaire outbreak revealed that the epidemic started in Guinea and spread to Sierra Leone and Liberia [28, 30, 74, 75]. During its rapid spread, base substitutions were identified that distinguished between early and late isolates [74], reviewed by [12]. To highlight the ability of EvoPrinter to identify subgroups, we illustrate how a multi-genome *EvoDifference* print, using the early isolate Gueckedou_GIN_C05_KJ660348.2_2014 genome as the input reference sequence, identified two subgroups within the Ebola/Zaire outbreak [marked by two identity SNPs at position 13,856 (A->G) and position 15,660 (T->C)], one represented by the Gueckedou subgroup (Guinea-1a), and a larger subgroup represented by the majority of Ebola/Zaire strains (S7A Fig). The accumulation of SNPs in Guinea-1a strains from Coyah and other locations illustrates the persistence of this early lineage over the course of the epidemic. The second identity SNP at position 15,660 (T->C), reinforces the hypothesis that the Coyah isolates, plus an isolate from Liberia, are part of the same early sublineage [12, 29, 30, 31].

Using a strain isolated during the later phase of the epidemic as the input reference sequence, identity SNPs were identified in the Sierra Leone-Guinea-3 sublineage [74, 76] (S7B Fig). Our analysis revealed an identity SNP at position 10218 (A->G), that marks isolates from Sierra Leone and Guinea. In addition, several Sierra Leone members of this subgroup are closely related to the reference sequence, while others are more distantly related, as seen by the presence of many SNP differences with the reference genome. Many sublineage A strains, described by [31] contain both an adenosine nucleotide at position 10218 and an additional A->G substution, at position 10273. Zaire strains with a G at position 10218 include the following: 1) all early sublineage Guinea-1a, 2) all Liberia strains, indicating their early origin during the course of the epidemic, 3) many Sierra Leone and Guinea isolates, both closely or distantly related to the reference sequence, and 4) a group of isolates from Guinea that contained an additional marker at position 10248 (T->C) (S7 Fig).

### Evidence for host cell A-to-I hyper-editing in Filoviruses

Host-cell adenosine deaminases that act on RNA (ADARs) modify RNAs by converting adenosine bases to inosines (for review, [53]). When ADARs edit a Filovirus replicative template, the viral polymerase interprets inosines as guanines, resulting in the negative stranded RNA genome having a cytosine instead of an uracil base at the modified or edited position. ADAR editing has been detected in both Marburg and Ebola isolates (for review, [44]).

**Fig 9:**
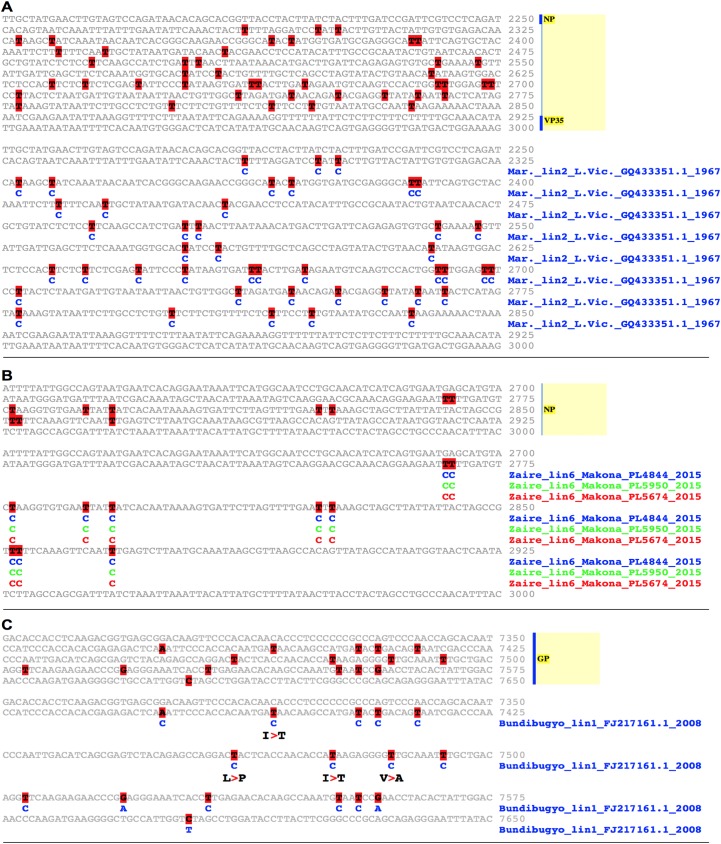
Filovirus A-to-I host cell hyper-editing detected with EvoDifference prints. One-on-one alignments that highlight clusters of T/U->C base changes within Marburg and Ebola (Zaire and Bundibugyo) genomes. (**A**) The Marburg_lin2_Popp_Cercopithecus _Human_Z29337.1_1967 reference sequence from bases 2,282 to 2,838 of the NP gene 3’ UTR and flanking sequence is aligned to the orthologous region of Marburg_lin2_LakeVictoria _GQ433353.1_2011. Note that all of the 40 base differences are T/U->C transformations, indicate that the Lake Victoria genome was most likely modified by host-cell RNA adenosine deaminases. (**B**) The Ebola Zaire_lin6_Kissidougou_GIN_C15_KJ660346_2014 VP40 3’UTR reference sequence, from bases 2742 to 2894 aligned with orthologous region from three different Zaire/Makona strains (listed in panel B). Given that the three lin6 strains have the same T/U->C base changes, host cell editing most likely occurred in an earlier member of this lineage. (**C**) Host cell A-to-I editing may contribute to the antigenic diversity of Filovirus spike proteins. Shown is the glycoprotein encoding mucin-like domain ORF of the Bundibugyo_lin2_DRC _112_KC545393.1_2012 isolate (bases 7,375 to 7,516) aligned to the orthologous region of the Bundibugyo_lin1_Uga_FJ217161.1_2008 genome. Note that four of the 13 T/U->C transitions result in amino acid changes (shown below the base substitutions). Color-coding is as described in Fig 1.

Whole-genome EvoDifference prints of related Marburg strains have revealed multiple T/U -> C base substitution clusters within non-coding regions and in protein encoding sequences of individual strains (Fig 9 and S8 Fig). For example, the intra-lineage comparison of the Marburg_lin2_Popp_Cercopithecus_Human_Z29337.1_1967 strain with the Marburg_lin2_LakeVictoria_GQ433353.1_2011 isolate identified a cluster of T -> C base differences within the NP gene 3’UTR and flanking VP35 intragenic region (Fig 9A). All 40 base differences in the 556 base non-coding region (bases 2,282 through 2,838) were identified as T/U -> C substitutions in the Lake Victoria strain. Examination of Marburg strains revealed examples of putative T/U -> C base editing in the VP35 and VP40 ORFs. Our analysis identified two strains with clusters of T/U -> C within the VP35 coding sequences. The first, found in Marburg_lin3_Ang_KM2601523.1 is illustrated in S8A Fig. The twenty-three T/U -> C base substitutions span 289 bases, 19 of which are in the intragenic region between the NP and VP35 coding regions; four additional substitutions are within the VP35 ORF and two of these resulted in nonsynonymous amino acid changes. The second example of A to I editing within the VP35 ORF was found in Marburg_lin9_Kenya_EU500826.1_1987 (S8B Fig). The T/U -> C substitutions resulted in 5 amino acid changes. Although overlapping in distribution, the substitutions in these lin3 and lin9 strains occurred at different positions.

We also identified evidence of T/U -> C substitutions in VP40 coding sequences. Within Marburg_lin9_Kenya_EU500828.1, fourteen T/U -> C base substitutions were identified (S8C Fig); 13 bases fell within the VP40 coding sequence. These substitutions resulted in four nonsynonymous amino acid changes. A second example identified in the VP40 coding sequence of Marburg_lin9_Kenya EU500826.1_1987 is illustrated in S8D Fig. These substitutions resulted in three amino acid changes.

Our search of Ebola genomes also uncovered clusters of T/U -> C base substitutions in Zaire and Bundibugyo strains. Within three Zaire_lin6_Port Loko_2015 isolates, we found identical T/U -> C patterns in the 3’ UTR of their NP genes (Fig 9B). The fact that the 3 isolates have the same T/U -> C substitutions indicates that the editing most likely occurred in a previous generation of this subgroup and was not a product of in vitro cell culture passage. In the Bundibugyo lineage, evidence of A-to-I editing was detected within the GP Mucin domain encoding sequence of the Bundibugyo_lin1_Uga_FJ217161.1_2008 strain (Fig 9C). Prior to full genomic sequencing, the GP gene from this Bundibugyo strain was sequenced from a patient serum-derived PCR product [21], indicating that the putative editing occurred in vivo. Of note, four of the 12 T/U -> C substitutions in the Bundibugyo_lin1_Uga_FJ217161.1_2008 mucin-like domain encoding sequence result in amino acid codon changes, suggesting that A-to-I editing may contribute to the antigenic diversity of the Filovirus spike proteins.

### Summary

The methodology and databases described here represent a new set of alignment tools for the rapid comparative analysis of a Flavivirus or Filovirus sequence. By superimposing alignment data of either one or up to hundreds of strains onto the user’s input sequence, uninterrupted readouts enable the following; 1) surveillance of lineage complexity within viral outbreaks, 2) the identification of unique base substitutions within the input sequence and/or database genomes, 3) the identification of recombinant strains, and 4) superimposed alignments highlight conserved sequence elements and allow for the identification of viral genomes that have been modified by host cell editing.

EvoPrinter should not be considered a stand-alone application for the analysis of Flavi or Filovirus evolution. We recommend that it’s search algorithms be used in conjunction with other tools that employ different sets of comparative analysis stratagies. For example, while EvoPrinter resolves sublineage markers and isolate-specific SNPs, other phylogenetic analysis programs provide information concerning lineage progression and diversification (e. g. [12–14]). Our strategy of detecting recombinants, using differential SNP patterns, is also complementary to other tools such as the multi-genome Recombination Detection Program that identifies recombinant fragments in graphic readouts [52]. When used together with these other tools, EvoPrinter should prove to be an important addition for the genetic surveillance of these evolving pathogens.

## Supporting information

S1 Fig Zika virus sublineages are resolved by their unique SNP patterns. (**A**) An EvoDifference print of the African Zika_KF383118.1_ Senegal_2001 strain, with nine database genomes listed in panel B (base color-coding as described in Fig 1). The sequence corresponds to 525 bases of the NS5-encoding region. The blue vertical bar indicates the sequence lines that were expanded to view database genome base differences in panel B. (**B**) Line numbers 9,025 and 9,175 were expanded to reveal sublineage specific SNP patterns. Note that the African isolates (from Central African Republic, Senegal and Nigeria) have similar, but non-identical SNP patterns, while the western hemisphere isolates share similar sequence differences with the input reference sequence and are different from the African lineages. Also note that the Brazil and Puerto Rico genomes differ from the Guatemalan isolates by a single base difference in sequence line number 9,150.
(PDF)

S2 Fig Yellow fever virus SNP differences visualized using a multi-genome EvoPrint. (**A**) An EvoPrint of YellowFever_GQ379162.1_Peru_2007 showing 600 bases of the non-structural NS3 protein coding region (codons 51 through 250) aligned to orthologous sequences from 15 South American and African Yellow Fever strains (listed in panel B). Unlike EvoDifference print readouts, in EvoPrints, black bases are identical in all genomes included in the analysis and gray bases indicate that one or more database genomes differ at that position from the input reference sequence. Note that the vertically stacked codons (achieved with 75 bases/line) reveal that the less-conserved bases mostly occupy codon wobble positions. The lack of wobble position conservation within most, but not all, of the codons indicates that the cumulative evolutionary divergence among the selected database genomes affords near base resolution of essential bases and their encoded amino acids. (**B**) Line number 5026 (showing the alignment details of bases 4952 to 5026) was expanded to show the different SNP patterns among the 15 database genomes (isolated from Brazil, Peru, Venezuela, Cote d’Ivoire, Senegal, Uganda and Ethiopia).
(PDF)

S3 Fig Distinguishing western hemisphere ZIKV sublineages. An EvoDifference print of Zika_KU365779.1_Brazil_2015 strain aligned with 41 other western hemisphere isolates resolves eight sublineages marked with identity SNPs. Three of the four Brazilian sublineages corresponds to those of Fig 4 and are presented here for comparison. Strains from Mexico fall into two classes, designated Central America 1 & 2. Puerto Rica sequences fall into a third class including strains from French Guiana and Suriname. Florida sequences fall into a separate group shared with two Dominica Republic strains. Note, the Dominica Republic isolates are heterogenous, as they share ID-SNPs with either Puerto Rico or Florida but not with both subgroups.
(PDF)

S4 Fig A Zika virus EvoDifference print identifies SNPs that distinguish Asian, Oceanian and South American subgroups. Pair-wise alignments between the Zika_KU955589.1_China_2016 (input reference sequence) with 18 Asian, Oceanian and South American strains. Shown, are 10 ID-SNP positions. The ID-SNP patterns resolve; 1) two distinct Chinese sublineages (Ch1 and Ch2), with second subgroup sharing many ID-SNPs with western hemisphere strains, 2) the Ch1 subgroup has unique ID-SNPs that distinguish it from western hemisphere strains, 3) Tonga and French Polynesian isolates represent an evolutionary intermediate position between the first Chinese subgroup and the Brazilian strains, and 4) The French Polynesian strains also share different sets of identity SNPs with the first Chinese subgroup (for example the KY447510.1 strain compared to the others) and the second Chinese subgroup shares ID-SNPs with western hemisphere strains. Note, the numbers following the Haiti, Brazil, Mexico and Dominican Republic strains indicate the number of same location isolates that have the same ID-SNP patterns.
(PDF)

S5 Fig One-on-one EvoDifference prints of a known Dengue2 recombinant virus highlight differential SNP patterns shared with its parental sublineages. Phylogenetic analysis of the Dengue2_AF100466.2_Venezuela_1990 (Mara4) strain has identified it as a recombinant that shares genome sequences with two different sublineages originating from Jamaica and Thailand [60]. Pairwise EvoDifference prints highlight SNP pattern differences between the parental lineages and the recombinant. Each alignment covers 10,682 bases. The reference (input) sequences are listed first, followed by the aligning database genome. (**A**) The Mara4 recombinant aligned to a member of the major parental lineage, Dengue2_M20558.1_Jamaica_1983. (**B**) Mara4 aligned to a member of the minor parental lineage, Dengue2_DQ181800.1_Thailand. (**C**) M20558.1_Jamaica (the major parental lineage member) aligned to DQ181800.1_Thailand (the minor parental lineage). The boxed sequence delimits the recombinant exchange region. The left side vertical color bar indicates positions of the different encoded proteins (Capsid, green, bases 1–366; Pre-Membrane, yellow, 367–900; Envelope, dark blue, 901–2400; NS1, gray, 2401–3426; NS2A, brown, 3427–4494; NS2B, green, 4494–5885; NS3, red, 5886–6345; NS4A, light blue, 6345–6726; 2K, black, 6727–6795; NS4B, tan, 6796–7501; NS5, orange, 7502–9975).
(PDF)

S6 Fig Abrupt changes in SNP densities and patterns identified in Zika African lineage EvoDifference prints indicate putative recombinant exchanges. (**A**) An EvoDifference print corresponding to 750 bases of the NS5 coding region from the Zika_KF383119_ Senegal_2001 strain generated with three other African genomes (listed in Panel B). The blue vertical bar highlights a 126 base sequence that contains a significantly higher SNP density relative to flanking sequences. (**B**) Line numbers 9,075 to 9,150 were expanded to reveal SNP patterns. Note the difference in SNP patterns and alternating changes in their density between the Zika_LC002520.1_Uganda_S.M._1947 and the Zika_KF383118.1_Senegal_2001 isolates, while the Zika_KF383115.1_C.A.R._1968 strain shares SNPs with both the Uganda and Senegal strains. Color-coding as described in Fig 1.
(PDF)

S7 Fig West African 2014–2015 Ebola/Zaire epidemic—early and late sublineage identity SNPs. EvoDifference prints of Ebola/Zaire strains from the West African epidemic of 2014 identifies subgroup identity SNPs within early and late Ebola epidemic isolates. The input reference strain for the early and late isolates is listed in panels A and B respectively. Sublineage designations were taken from [31] and [32]. Numbers in the right column following database strain names represent number of bases by which they differ from the input reference genome and were regrouped from the initial alignment to highlight SNP identity subgroups. (**A**) Using a strain from the earliest lineage identified during the epidemic, Guinea-1a (reference input: Gueckedou_GIN_C05_KJ660348.2_2014) a multi-genome EvoDifference print was generated using isolates from both early and late stages of the epidemic as listed. Sublineage identity SNPs differentiated early and late isolates at bases 13856 and 15660. Illustrated are 18 isolates with with A and T at these positions, respectively, while seven isolates are shown that had the G and C, respectively, at these positions, that are also found in most other late isolates. (**B**) Using an isolate from a late sublineage, designated Sierra Leone-Guinea-3 (input reference sequence Zaire_lin6_Makona_ SLE_G3856.1_2014), a multi-genome EvoDifference print was generated using isolates from both early and late stages of the epidemic as listed. The readout identifies a sublineage marker at base position 10218 as well as sublineage identity SNPs at 10248 and 10273 positions. The database entries are also grouped according to their country of origin. The reference sequence, with an A nucleotide at position 10218, is distinct from Guinea-1a lineage, which was generated early in the epidemic, with all other genomes having a G at position 10218. All isolates with an A at position 10218 are from Sierra Leone (total of 65 in the database) or Guinea (total of 19 in the database) while the Liberian strains have a G at position 10218 indicating their origin early during the course of the epidemic. Many sublineage-A sequences are highly diverged from the input reference sequence yet share with the input an A at position 10218 (total of 30 in the database). Isolates with a G at position 10218 include 13 from Sierra Leone and 39 from Guinea (including 9 with a C at position 10248).
(PDF)

S8 Fig A-to-I host cell hyper-editing in Marburg virus open reading frames detected using EvoDifference prints. One-on-one alignments of related Marburg strains highlight clusters of T/U->C base changes within the VP35 and VP40 ORFs. **(A1)** The Marburg_lin3_Ang_LakeVictoria_1381_DQ447654.1_2005 reference sequence from bases 2550 to 3300 including a portion of intragenic region between NP and VP35 and the start of the VP35 open reading frame (marked by the green carat) aligned with Marburg_lin3_Ang_KM261523.1_2005. The four expanded sequence lines reveals a cluster of 23 T/U -> C base substitutions extending 289 bases, 19 of which are in the intragenic region between NP and VP35 coding regions; four additional substitutions are found within the VP35 ORF. (**A2** and **A3**) Of these four substitutions, two resulted in nonsynonymous amino acid changes, illustrated using the *EvoPrinter* translation utility. (**B1**) The Marburg_lin9_Kenya_LakeVictoria_Ravn_R1_EU500827.1_1987 reference sequence from bases 2776 to 3375 aligned with Marburg_lin9_Kenya EU500827.1_1987. Two sequence lines of the VP-35 coding sequence, that exhibit base substitutions (red highlight), have been expanded to reveal the substitution (T to C) in the Kenya isolate. All substitutions occurred in the open reading frame (the methionine-encoding ATG start site is marked with the green carat). (**B2** and **B3**) As a result of the A to I editing, five nonsynonymous changes occurred in the encoded amino acids. (**C1**) The Marburg_lin9_DRG_DQ447652.1_1999 reference sequence from bases 4376 to 4875 of the region of the VP40 coding sequence is aligned to the orthologous region of Marburg_lin9_Kenya_EU500827.1_1987. Three sequence lines that exhibit base substitutions (red highlight) have been expanded to reveal the substitution (T->C) in the Kenya isolate. All but the first occurred in the open reading frame (the methionine-encoding ATG start site is marked with the green carat). (**C2** and **C3**) As a result of the (T->C) substitutions nonsynonymous changes occurred in five encoded amino acids. (**D1**) The Marburg_lin9_Kenya_LakeVictoria_Ravn_R1_EU500827.1_1987 reference sequence from bases 4365 to 5025 aligned with Marburg_lin9_Kenya_EU500826.1_1987 to reveal T/U -> C base substitutions in a VP40 coding sequence. Two sequence lines that exhibit the base substitutions (red highlight) have been expanded to reveal the substitutions in the Marburg_lin9_Kenya EU500827.1_1987 isolate. All T to C substitutions in this cluster occurred in the open reading frame (ATG start site is marked with the green carat). (**D2** and **D3**) As a result of the A to I editing, three nonsynonymous changes were detected that result in amino acids.
(PDF)
