# Supplementary material for: Flavivirus and Filovirus EvoPrinters: New alignment tools for the comparative analysis of viral evolution
Source: PLoS Negl Trop Dis. 2017 Jun 16;11(6):e0005673. doi: 10.1371/journal.pntd.0005673 (PMC5489223; doi:10.1371/journal.pntd.0005673)
Supplement: S1 Fig — (A) An EvoDifference print of the African Zika_KF383118.1_ Senegal_2001 strain, with nine database genomes listed in panel B (base color-coding as described in Fig 1). The sequence corresponds to 525 bases of the NS5-encoding region. The blue vertical bar indicates the sequence lines that were expanded to view database genome base differences in panel B. (B) Line numbers 9,025 and 9,175 were expanded to reveal sublineage specific SNP patterns. Note that the African isolates (from Central African Republic, Senegal and Nigeria) have similar, but non-identical SNP patterns, while the western hemisphere isolates share similar sequence differences with the input reference sequence and are different from the African lineages. Also note that the Brazil and Puerto Rico genomes differ from the Guatemalan isolates by a single base difference in sequence line number 9,150. (PDF) [file pntd.0005673.s001.pdf]

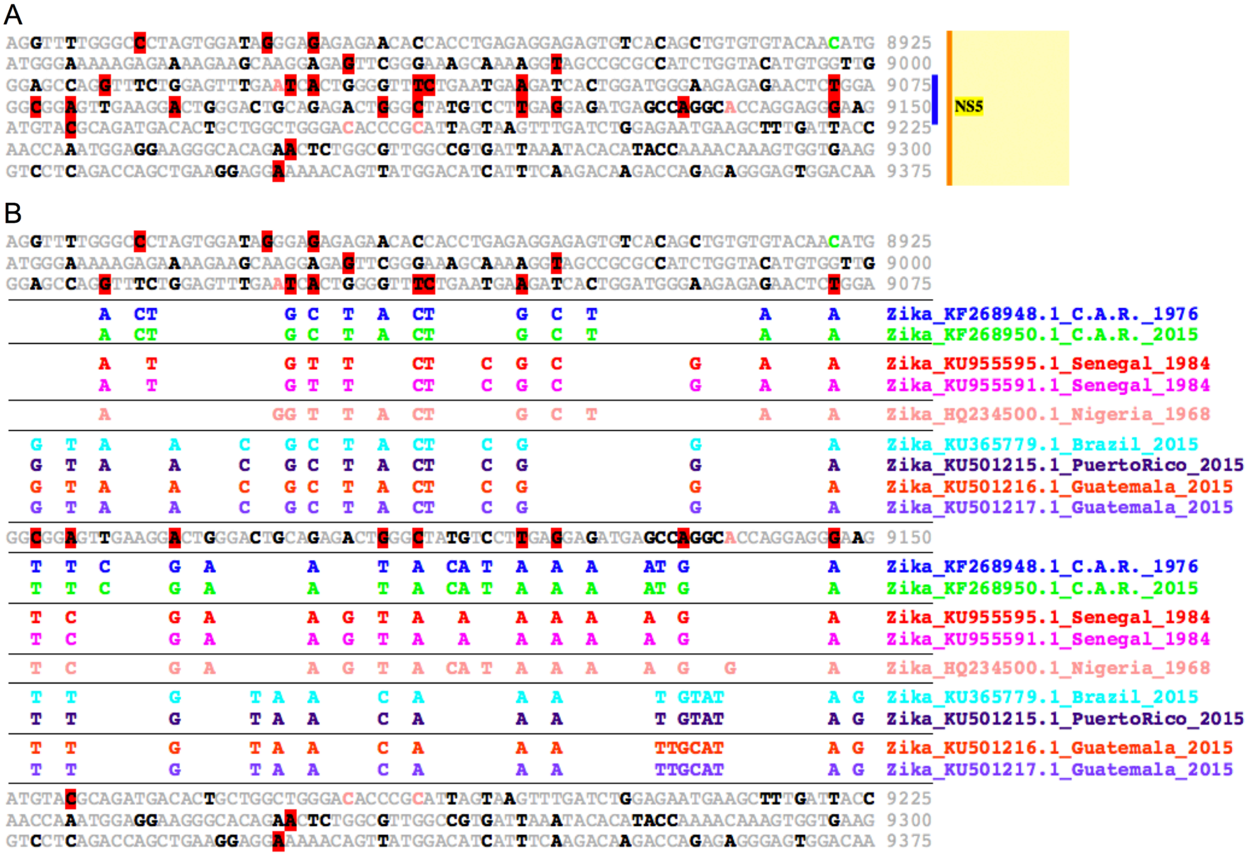


**S1_fig.pdf  Zika virus sublineages are resolved by their shared SNP patterns**

(**A**) An *EvoDifference* print of the African *Zika_KF383118.1_ Senegal_2001* strain, with nine database genomes listed in panel B (base color-coding as described in Figure 1). The sequence corresponds to 525 bases of the NS5-encoding region. The blue vertical bar indicates the sequence lines that were expanded to view database genome base differences in panel B. (**B**) Line numbers 9,025 and 9,175 were expanded to reveal sublineage specific SNP patterns. Note that the African isolates (from Central African Republic, Senegal and Nigeria) have similar, but non-identical SNP patterns, while the western hemisphere isolates share similar sequence differences with the input reference sequence and are different from the African lineages. Also note that the Brazil and Puerto Rico genomes differ from the Guatemalan isolates by a single base difference in sequence line number 9,150.
