# Supplementary material for: Flavivirus and Filovirus EvoPrinters: New alignment tools for the comparative analysis of viral evolution
Source: PLoS Negl Trop Dis. 2017 Jun 16;11(6):e0005673. doi: 10.1371/journal.pntd.0005673 (PMC5489223; doi:10.1371/journal.pntd.0005673)
Supplement: S2 Fig — (A) An EvoPrint of YellowFever_GQ379162.1_Peru_2007 showing 600 bases of the non-structural NS3 protein coding region (codons 51 through 250) aligned to orthologous sequences from 15 South American and African Yellow Fever strains (listed in panel B). Unlike EvoDifference print readouts, in EvoPrints, black bases are identical in all genomes included in the analysis and gray bases indicate that one or more database genomes differ at that position from the input reference sequence. Note that the vertically stacked codons (achieved with 75 bases/line) reveal that the less-conserved bases mostly occupy codon wobble positions. The lack of wobble position conservation within most, but not all, of the codons indicates that the cumulative evolutionary divergence among the selected database genomes affords near base resolution of essential bases and their encoded amino acids. (B) Line number 5026 (showing the alignment details of bases 4952 to 5026) was expanded to show the different SNP patterns among the 15 database genomes (isolated from Brazil, Peru, Venezuela, Cote d’Ivoire, Senegal, Uganda and Ethiopia). (PDF) [file pntd.0005673.s002.pdf]

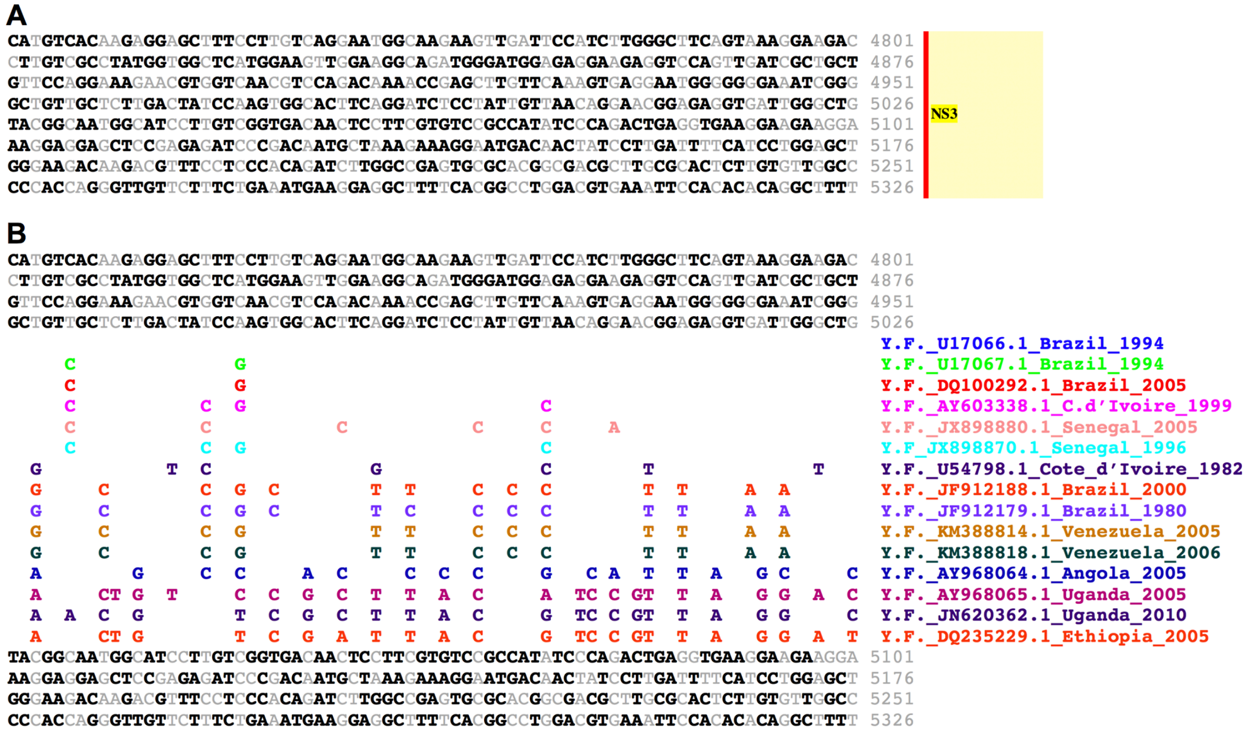

S2_fig.pdf Yellow Fever virus SNP differences visualized with a multi-genome *EvoPrint*

(**A**) An *EvoPrint* of the *YellowFever_GQ379162.1_Peru_2007* strain showing 600 bases of the non-structural NS3 protein coding region (codons 51 through 250) aligned to orthologous sequences from 15 South American and African Yellow Fever strains (listed in panel B). Unlike *EvoDifference* print readouts, in *EvoPrints,* black bases are identical in all genomes included in the analysis and gray bases indicate that one or more database genomes differ at that position from the input reference sequence. Note that the vertically stacked codons (achieved with 75 bases/line) reveal that the less-conserved bases mostly occupy codon wobble positions. The lack of wobble position conservation within most, but not all, of the codons indicates that the cumulative evolutionary divergence among the selected database genomes affords near base resolution of essential bases and their conserved encoded amino acids. (**B**) Line number 5026 (showing the alignment details of bases 4952 to 5026) was expanded to show the different SNP patterns among the 15 database genomes (isolated from Brazil, Peru, Venezuela, Cote d'Ivoire, Senegal, Uganda and Ethiopia).
