# Supplementary material for: Flavivirus and Filovirus EvoPrinters: New alignment tools for the comparative analysis of viral evolution
Source: PLoS Negl Trop Dis. 2017 Jun 16;11(6):e0005673. doi: 10.1371/journal.pntd.0005673 (PMC5489223; doi:10.1371/journal.pntd.0005673)
Supplement: S3 Fig — An EvoDifference print of Zika_KU365779.1_Brazil_2015 strain aligned with 41 other western hemisphere isolates resolves eight sublineages marked with identity SNPs. Three of the four Brazilian sublineages corresponds to those of Fig 4 and are presented here for comparison. Strains from Mexico fall into two classes, designated Central America 1 & 2. Puerto Rica sequences fall into a third class including strains from French Guiana and Suriname. Florida sequences fall into a separate group shared with two Dominica Republic strains. Note, the Dominica Republic isolates are heterogenous, as they share ID-SNPs with either Puerto Rico or Florida but not with both subgroups. (PDF) [file pntd.0005673.s003.pdf]

1461	1890	3552	3578	3975	5181	5320	6615	6657	6711	6723	6969	7079	9228	9297	Input reference genome: Zika_KU365779.1_Brazil_2015
AA**A**GT	AC**T**GC	GA**C**CA	AT**T**CT	GA**T**AA	TT**G**CA	GA**G**GC	AT**T**AT	TT**C**TT	AC**T**CT	AG**C**GC	GG**G**GC	AC**T**TC	CG**T**AT	CT**G**GA	
															Brazil 3
															KU365779.1_Brazil_2015
															KU707826.1_Brazil_2015
															KU365777.1_Brazil_2015
															Brazil 2
G													C		KU940224.1_Brazil_2015
G													C		KU940228.1_Brazil_2015
G													C		KX520666.1_Brazil_2015
															Brazil 1
G	C	T						T					C		KX197192.1_Brazil_2015
G	C	T						T			A		C		KX811222.1_Brazil_2015
G	C	T						T			A		C		KU729217.2_Brazil_2015
															Brazil 4
G	C	T		C		A		T					C		KU991811.1_Brazil_2016
G	C	T		C		A		T					C		KU497555.1_Brazil_2015
G	C	T				A		T					C		KU729218.1_Brazil_2015
															Central America 1
G	C	T		C		A		T					C		KX087102.1_Columbia_2015
G	C	T		C		A		T					C		KX247646.1_Columbia_2016
G	C	T		C		A		T					C		KU820897.1_Columbia_2015
G	C	T		C		A		T					C		KU647676.1_Martinique_2015
G	C	T		C		A		T					C		KU922960.1_Mexico_2016
G	C	T		C		A		T					C		KU922923.1_Mexico_2016
G	C	T		C		A		T					C		KX198135.1_Panama_2016
G	C	T		C		A		T					C		KX156775.1_Panama_2015
G	C	T		C		A		T					C		KX156776.1_Panama_2015
G	C	T		C		A		T					C		KX702400.1_Venezuela_2016
															Central America 2
G	C	T			A			T	C	T					KU870645.1_Guatemala_2016
G	C	T			A			T	C	T					KU501216.1_Guatemala_2015
G	C	T			A			T	C	T					KU501217.1_Guatemala_2015
G	C	T			A			T	C	T					KX262887.1_Honduras_2016
G	C	T			A			T	C	T					KX694534.1_Honduras_2015
G	C	T			A			T	C	T					KX446950.1_Mexico_2016
G	C	T			A			T	C	T					KX247632.1_Mexico_2015
G	C	T			A			T	C	T					KX446951.1_Mexico_2016
															South America & Caribbean
N			C				A					C			KX766028.1_Dom.Republic_2016
			C				A					C			KU758877.1_FrenchGuiana_2015
			C				A					C			KU501215.1_PuertoRico_2015
			C				A					C			KX377337.1_PuertoRico_2016
			C				A					C			KX601168.1_PuertoRico_2015
			C				A					C			KU937936.1_Suriname_2016
															Dom.Republic & Florida
G								T			A		C	A	KU853012.1_Dom.Republic_2016
G								T			A		C	A	KU853013.1_Dom.Republic_2016
G								T			A		C	A	KX842449.1_Florida_2016
G								T			A		C	A	KX838905.1_Florida_Aedes_2016
G								T			A		C	A	KX832731.1_Florida_2016

S3_fig.pdf Identity SNPs resolve different western hemisphere ZIKV sublineages

An *EvoDifference* print of the *Zika_KU365779.1_Brazil_2015* strain aligned with 41 other western hemisphere isolates identifies eight sublineages distinguished by ID-SNPs. Three of the four Brazilian sublineages corresponds to those of figure 3 and are presented here for comparison. Strains from Mexico fall into two classes, designated Central America 1 & 2. Puerto Rica sequences fall into a third class including strains from French Guiana and Suriname. Florida sequences fall into a separate group shared with two Dominica Republic strains. Note, the Dominica Republic isolates are heterogenous, as they share ID-SNPs with either Puerto Rico or Florida but not with both subgroups. Only bases that disagree with the input reference sequences are shown.
