# Supplementary material for: Flavivirus and Filovirus EvoPrinters: New alignment tools for the comparative analysis of viral evolution
Source: PLoS Negl Trop Dis. 2017 Jun 16;11(6):e0005673. doi: 10.1371/journal.pntd.0005673 (PMC5489223; doi:10.1371/journal.pntd.0005673)
Supplement: S4 Fig — Pair-wise alignments between the Zika_KU955589.1_China_2016 (input reference sequence) with 18 Asian, Oceanian and South American strains. Shown, are 10 ID-SNP positions. The ID-SNP patterns resolve; 1) two distinct Chinese sublineages (Ch1 and Ch2), with second subgroup sharing many ID-SNPs with western hemisphere strains, 2) the Ch1 subgroup has unique ID-SNPs that distinguish it from western hemisphere strains, 3) Tonga and French Polynesian isolates represent an evolutionary intermediate position between the first Chinese subgroup and the Brazilian strains, and 4) The French Polynesian strains also share different sets of identity SNPs with the first Chinese subgroup (for example the KY447510.1 strain compared to the others) and the second Chinese subgroup shares ID-SNPs with western hemisphere strains. Note, the numbers following the Haiti, Brazil, Mexico and Dominican Republic strains indicate the number of same location isolates that have the same ID-SNP patterns. (PDF) [file pntd.0005673.s004.pdf]

Input Sequence: ***Zika_KU955589.1_China_2016***

1408	9740	9367	7940	2587	8341	366	9471	2166	4552		
AAGGT	CACTT	AAGAC	CCATG	TGTGG	CCCAG	TAATG	GAGTA	CAGGA	GGAGC		
										Zika_KU820899.2_China_2016	Ch 1
										Zika_KU963796.1_China_2016	Ch 1
										Zika_KX117076.1_China_2016	Ch 1
										Zika_KU866423.1_China_2016	Ch 1
								A	T	Zika_KX806557.1_Tonga_2016	
						G	A	A	T	Zika_KJ776791.1_F.Polynesia_2013	
						G	A	A	T	Zika_KX369547.1_F.Polynesia_2013	
						G	A	A	T	Zika_KX447515.1_F.Polynesia_2014	
				C	T	G	A	A	T	Zika_KX447510.1_F.Polynesia_2014	
			G	C	T	G	A	A	T	Zika_KX051563.1_Haiti_2016 (2)	
		A	G	C	T	G	A	A	T	Zika_KU527068.1_Brazil_2015 (14)	
		A	G	C	T	G	A	A	T	Zika_KU922960.1_Mexico_2016 (2)	
		A	G	C	T	G	A	A	T	Zika_KU853012.1_D.Republic_2016 (2)	
A	T	A	G	C	T	G	A	A	T	Zika_KU820898.1_China_2016	Ch 2
A	T	A	G	C	T	G	A	A	T	Zika_KX056898.1_China_2016	Ch 2
A	T	A	G	C	T	G	A	A	T	Zika_KU740184.2_China_2016	Ch 2
A	T	A	G	C	T	G	A	A	T	Zika_KU955590.1_China_2016	Ch 2

S4_fig.pdf ID-SNPs resolve different Asian, Oceanian and South American ZIKV sublineages

Pair-wise alignments between the *Zika_KU955589.1_China_2016* (input reference sequence) with 18 Asian, Oceanian and South American strains. Shown, are 10 ID-SNP positions. The ID-SNP patterns resolve; 1) two distinct Chinese sublineages (Ch1 and Ch2), with second subgroup sharing many ID-SNPs with western hemisphere strains, 2) the Ch1 subgroup has unique ID-SNPs that distinguish it from western hemisphere strains, 3) Tonga and French Polynesian isolates represent an evolutionary intermediate position between the first Chinese subgroup and the Brazilian strains, and 4) The French Polynesian strains also share different sets of identity SNPs with the first Chinese subgroup (for example the *KY447510.1* strain compared to the others) and the second Chinese subgroup shares ID-SNPs with western hemisphere strains. Note, the numbers following the Haiti, Brazil, Mexico and Dominican Republic strains indicate the number of same location isolates that have the same ID-SNP patterns.
