# Supplementary material for: Flavivirus and Filovirus EvoPrinters: New alignment tools for the comparative analysis of viral evolution
Source: PLoS Negl Trop Dis. 2017 Jun 16;11(6):e0005673. doi: 10.1371/journal.pntd.0005673 (PMC5489223; doi:10.1371/journal.pntd.0005673)
Supplement: S5 Fig — Phylogenetic analysis of the Dengue2_AF100466.2_Venezuela_1990 (Mara4) strain has identified it as a recombinant that shares genome sequences with two different sublineages originating from Jamaica and Thailand [60]. Pairwise EvoDifference prints highlight SNP pattern differences between the parental lineages and the recombinant. Each alignment covers 10,682 bases. The reference (input) sequences are listed first, followed by the aligning database genome. (A) The Mara4 recombinant aligned to a member of the major parental lineage, Dengue2_M20558.1_Jamaica_1983. (B) Mara4 aligned to a member of the minor parental lineage, Dengue2_DQ181800.1_Thailand. (C) M20558.1_Jamaica (the major parental lineage member) aligned to DQ181800.1_Thailand (the minor parental lineage). The boxed sequence delimits the recombinant exchange region. The left side vertical color bar indicates positions of the different encoded proteins (Capsid, green, bases 1–366; Pre-Membrane, yellow, 367–900; Envelope, dark blue, 901–2400; NS1, gray, 2401–3426; NS2A, brown, 3427–4494; NS2B, green, 4494–5885; NS3, red, 5886–6345; NS4A, light blue, 6345–6726; 2K, black, 6727–6795; NS4B, tan, 6796–7501; NS5, orange, 7502–9975). (PDF) [file pntd.0005673.s005.pdf]

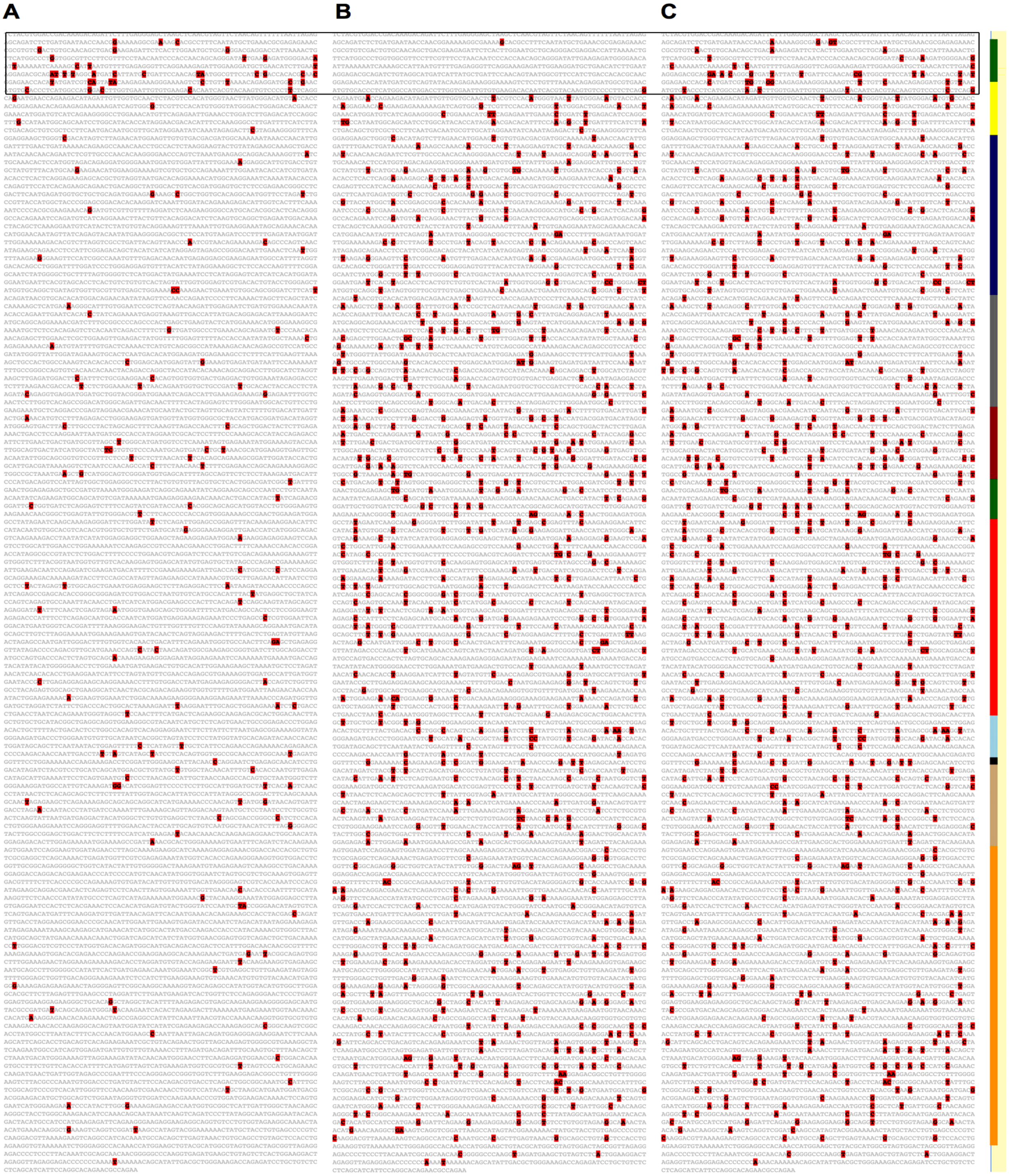

S5_fig.pdf One-on-one *EvoDifference* prints of a known Dengue2 recombinant virus highlight differential SNP patterns shared with its parental sublineages

Phylogenetic analysis of the *Dengue2_AF100466.2_Venezuela_1990* (Mara4) strain has identified it as a recombinant that shares genome sequences with two different sublineages

originating from Jamaica and Thailand [60]. Pairwise *EvoDifference* prints highlight SNP pattern differences between the parental lineages and the recombinant. Each alignment covers 10,682 bases. The reference (input) sequences are listed first, followed by the aligning database genome. (**A**) The Mara4 recombinant aligned to a member of the major parental lineage, *Dengue2_M20558.1_Jamaica_1983*. (**B**) Mara4 aligned to a member of the minor parental lineage, *Dengue2_DQ181800.1_Thailand*. (**C**) *M20558.1_Jamaica* (the major parental lineage member) aligned to *DQ181800.1_Thailand* (the minor parental lineage). The boxed sequence delimits the recombinant exchange region. The left side vertical color bar indicates positions of the different encoded proteins (Capsid, green, bases 1-366; Pre-Membrane, yellow, 367-900; Envelope, dark blue, 901-2400; NS1, gray, 2401-3426; NS2A, brown, 3427-4494; NS2B, green, 4494-5885; NS3, red, 5886-6345; NS4A, light blue, 6345-6726; 2K, black, 6727-6795; NS4B, tan, 6796-7501; NS5, orange, 7502-9975).
