# Supplementary material for: Flavivirus and Filovirus EvoPrinters: New alignment tools for the comparative analysis of viral evolution
Source: PLoS Negl Trop Dis. 2017 Jun 16;11(6):e0005673. doi: 10.1371/journal.pntd.0005673 (PMC5489223; doi:10.1371/journal.pntd.0005673)
Supplement: S6 Fig — (A) An EvoDifference print corresponding to 750 bases of the NS5 coding region from the Zika_KF383119_ Senegal_2001 strain generated with three other African genomes (listed in Panel B). The blue vertical bar highlights a 126 base sequence that contains a significantly higher SNP density relative to flanking sequences. (B) Line numbers 9,075 to 9,150 were expanded to reveal SNP patterns. Note the difference in SNP patterns and alternating changes in their density between the Zika_LC002520.1_Uganda_S.M._1947 and the Zika_KF383118.1_Senegal_2001 isolates, while the Zika_KF383115.1_C.A.R._1968 strain shares SNPs with both the Uganda and Senegal strains. Color-coding as described in Fig 1. (PDF) [file pntd.0005673.s006.pdf]

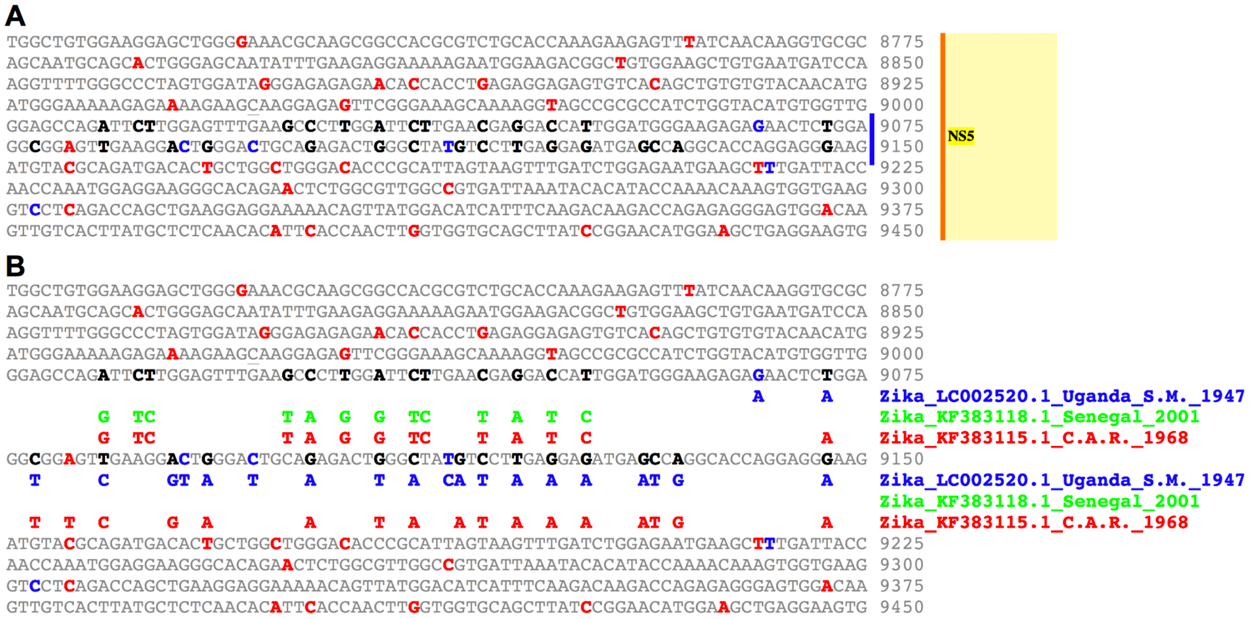

S6_fig.pdf Abrupt changes in SNP profiles identified in an African lineage ZIKV *EvoDifference* print indicate putative recombinant exchanges

(**A**) An *EvoDifference* print corresponding to 750 bases of the NS5 coding region from the *Zika_KF383119_ Senegal_2001* strain generated with three other African genomes (listed in Panel B). The blue vertical bar highlights a 126 base sequence that contains a higher SNP density relative to flanking sequences. (**B**) Line numbers 9,075 to 9,150 were expanded to reveal SNP patterns. Note the difference in SNP patterns and alternating changes in their density between the *Zika_LC002520.1_Uganda_S.M._1947* and the *Zika_KF383118.1 _Senegal_2001* isolates, while the *Zika_KF383115.1_C.A.R._1968* strain shares SNPs with both the Uganda and Senegal strains. Color-coding as described in Figure 1.
