# Supplementary material for: Flavivirus and Filovirus EvoPrinters: New alignment tools for the comparative analysis of viral evolution
Source: PLoS Negl Trop Dis. 2017 Jun 16;11(6):e0005673. doi: 10.1371/journal.pntd.0005673 (PMC5489223; doi:10.1371/journal.pntd.0005673)
Supplement: S7 Fig — EvoDifference prints of Ebola/Zaire strains from the West African epidemic of 2014 identifies subgroup identity SNPs within early and late Ebola epidemic isolates. The input reference strain for the early and late isolates is listed in panels A and B respectively. Sublineage designations were taken from [31] and [32]. Numbers in the right column following database strain names represent number of bases by which they differ from the input reference genome and were regrouped from the initial alignment to highlight SNP identity subgroups. (A) Using a strain from the earliest lineage identified during the epidemic, Guinea-1a (reference input: Gueckedou_GIN_C05_KJ660348.2_2014) a multi-genome EvoDifference print was generated using isolates from both early and late stages of the epidemic as listed. Sublineage identity SNPs differentiated early and late isolates at bases 13856 and 15660. Illustrated are 18 isolates with with A and T at these positions, respectively, while seven isolates are shown that had the G and C, respectively, at these positions, that are also found in most other late isolates. (B) Using an isolate from a late sublineage, designated Sierra Leone-Guinea-3 (input reference sequence Zaire_lin6_Makona_ SLE_G3856.1_2014), a multi-genome EvoDifference print was generated using isolates from both early and late stages of the epidemic as listed. The readout identifies a sublineage marker at base position 10218 as well as sublineage identity SNPs at 10248 and 10273 positions. The database entries are also grouped according to their country of origin. The reference sequence, with an A nucleotide at position 10218, is distinct from Guinea-1a lineage, which was generated early in the epidemic, with all other genomes having a G at position 10218. All isolates with an A at position 10218 are from Sierra Leone (total of 65 in the database) or Guinea (total of 19 in the database) while the Liberian strains have a G at position 10218 indicating their origin early dur [file pntd.0005673.s007.pdf]

A *Zaire_lin6_Gueckedou_GIN_C05_KJ660348.2_2014*

```
                                                     13856
GGTGACAATCAGTGCATTACCGTTTTATCAGTCTTCCCCTTAGAGACTGATGCAGACGAGCAGGAACAGAGCGCC 13875
                                                                            Gueckedou_GIN_WPG_C05_KP096420.1_2014        3
                                                                            Gueckedou_GIN_WPG_C07_KP096421.1_2014        3
                                                                            Gueckedou_GIN_C07_KJ660347.2_2014            4
                                                                            Makona_LBR_EM_079450_KR817194.1_2014         7
                                                                            Makona_GIN_Kouroussa_531_KR534510.1_2        10
                                                                            Makona_GIN_Siguiri_517_KR534509.1_2014       11
                                                                            Makona_GIN_Coyah_1278_KR534544.1_2014        15
                                                                            Makona_GIN_Conakry_1193_KR534537.1_2014      15
                                                                            Makona_GIN_Coyah_1689_KR534572.1_2014        15
                                                                            Makona_GIN_Coyah_1327_KR534591.1_2014        16
                                                                            Makona_GIN_Coyah_1436_KR534558.1_2014        16
                                                                            Makona_GIN_Coyah_1277_KR534543.1_2014        17
                                                                            Makona_GIN_Coyah_1316_KR534546.1_2014        17
                                                                            Makona_GIN_Coyah_1320_KR534590.1_2014        17
                                                                            Makona_GIN_Conakry_1129_KR534580.1_2014      18
                                                                            Makona_GIN_Kindia_1648_KR534569.1_2014       20
                                                                            sublinGuinea_Makona_SLE_PL5294_Kambia_2015   26

                                                       G                    Kissidougou_GIN_C15_KJ660346.2_2014          5
                                                       G                    Makona_SLE_EM095_KM034550.1_2014             7
                                                       G                    Gueckedou_GIN_WPG_C15_KP096422.1_2014        10
                                                       G                    Makona_LBR_EM_080064_KR817231.1_2014         10
                                                       G                    Makona_LBR_EM_080066_KR817233.1_2014         10
                                                       G                    Makona_LBR_10237_KT725275.1_2014             11
                                                       G                    Gueckedou_GIN_Conacry_192_KP342330.1_2014    12

                                                          15660
TATAATCGTGCTCACCTTCATCTAACTAAGTGTTGCACCCGGGAGGTACCAGCTCAGTATTTAACATACACATCT 15675
                                                                            Gueckedou_GIN_WPG_C05_KP096420.1_2014        3
                                                                            Gueckedou_GIN_WPG_C07_KP096421.1_2014        3
                                                                            Gueckedou_GIN_C07_KJ660347.2_2014            4
                                                                            Makona_LBR_EM_079450_KR817194.1_2014         7
                                                              G             Makona_GIN_Kouroussa_531_KR534510.1_2014     10
                                                              G             Makona_GIN_Siguiri_517_KR534509.1_2014       11
                                                                            Makona_GIN_Conakry_1193_KR534537.1_2014      15
                                                                            Makona_GIN_Coyah_1278_KR534544.1_2014        15
                                                                            Makona_GIN_Coyah_1689_KR534572.1_2014        15
                                                                            Makona_GIN_Coyah_1327_KR534591.1_2014        16
                                                                            Makona_GIN_Coyah_1436_KR534558.1_2014        16
                                                                            Makona_GIN_Coyah_1277_KR534543.1_2014        17
                                                                            Makona_GIN_Coyah_1316_KR534546.1_2014        17
                                                                            Makona_GIN_Coyah_1320_KR534590.1_2014        17
                                                                            Makona_GIN_Conakry_1129_KR534580.1_2014      18
                                                                            Makona_GIN_Kindia_1648_KR534569.1_2014       20
                                                                            sublinGuinea_Makona_SLE_PL5294_Kambia_2015   26

                                                           C                Kissidougou_GIN_C15_KJ660346.2_2014          5
                                                           C                Makona_SLE_EM095_KM034550.1_2014             7
                                                           C                Gueckedou_GIN_WPG_C15_KP096422.1_2014        10
                                                           C                Makona_LBR_EM_080064_KR817231.1_2014         10
                                                           C                Makona_LBR_EM_080066_KR817233.1_2014         10
                                                           C                Makona_LBR_10237_KT725275.1_2014             11
                                                           C                Gueckedou_GIN_Conacry_192_KP342330.1_2014    12
```

B *Zaire_lin6_Makona_SLE_G3856.1_2014*

```
                 10218                         10248                    10273
CGAACATTACTTTGAGCACCCTCACAATTAAAAAATAAGAGCGTCGTTCCAACAATCGAGCGCAAGGTTACAAGG 10275
```

10218	10248	10273	Strain	
			Makona_SLE_EM124.1_KM233045.1_2014	1
			Makona_SLE_EM104_KM233035.1_2014	2
			Makona_SLE_G3713.4_2014	2
			Makona_SLE_G3724_2014	2
			Makona_SLE_Kenema_KR105268_2014	7
			Makona_SLE_PL5801_Kambia_2015	15
			Makona_SLE_PL6158_Kambia_2015	16
			Makona_SLE_PL6070_Kambia_2015	21
			Gueckedou_GIN_Conacry_192_KP342330.1_2014	3
			Makona_GIN_Conakry_653_KR534514.1_2014	3
			Makona_GIN_Conakry_976_KR534525.1_2014	3
			Makona_GIN_Conakry_1027_KR534577.1_2014	7
			Makona_GIN_Conakry_1651_KR534570.1_2014	7
			Makona_GIN_Conakry_684_KR534517.1_2014	7
		G	sublinA_Makona_SLE_KT5382_W.Rural_2015	11
		G	sublinA_Makona_SLE_MK2371_Bombali_2015	11
		G	sublinA_Makona_SLE_KT5788_W.Rural_2015	12
		G	sublinA_Makona_SLE_MK2342_Bombali_2015	27
		G	sublinA_Makona_SLE_MK2369_Bombali_2015	27
		G	sublinA_Makona_SLE_MK2362_Bombali_2015	37
G			Makona_LBR_10237_KT725275.1_2014	2
G			Makona_LBR_10187_KT725392.1_2014	3
G			Makona_LBR_10241_KT725262.1_2014	3
G			Makona_LBR_10250_KT725360.1_2014	3
G			Makona_LBR_0284_KT725328.1_2014	25
G			Makona_LBR_10051_KT725368.1_2014	28
G			Makona_LBR_10127_KT725272.1_2014	38
G			Makona_SLE_G3823_2014	3
G			Makona_SLE_EM096_KM034551.1_2014	4
G			Makona_SLE_EM121_KM233044.1_2014	13
G			Makona_SLE_G3769.4_2014	13
G			Makona_SLE_G3810.1_2014	13
G			Makona_SLE_G3831_2014	13
G			Makona_GIN_Conakry_768_KR534588.1_2014	6
G			Kissidougou_GIN_C15_KJ660346.2_2014	8
G			Makona_GIN_Conakry_1120_KR534535.1_2014	8
G			Makona_GIN_Conakry_1480_KR534561.1_2014	8
G			Gueckedou_GIN_WPG_C07_KP096421.1_2014	14
G			Gueckedou_GIN_WPG_C05_KP096420.1_2014	16
G			Makona_GIN_Coyah_1374_KR534556.1_2014	18
G			Makona_GIN_Kindia_1648_KR534569.1_2014	20
G	C		Makona_GIN_Dubreka_789_KR534523.1_2014	8
G	C		Makona_GIN_Conakry_1342_KR534552.1_2014	9
G	C		Makona_GIN_Conakry_1210_KR534539.1_2014	10
G	C		Makona_GIN_Conakry_1340_KR534551.1_2014	10
G	C		Makona_GIN_Conakry_1561_KR534563.1_2014	11
G	C		Makona_GIN_Nzeerekore_1622_KR534567.1_2014	13
G	C		Makona_GIN_Coyah_1436_KR534558.1_2014	23

S7_fig.pdf West African 2014-2015 Ebola/Zaire epidemic - early and late sublineage identity SNPs.

EvoDifference prints of Ebola/Zaire strains from the West African epidemic of 2014 identifies subgroup identity SNPs within early and late Ebola epidemic isolates. The input reference strain for the early and late isolates is listed in panels A and B respectively. Sublineage designations were taken from [31] and [32]. Numbers in the right column following database strain names represent number of bases by which they differ from the input reference genome and were regrouped from the initial alignment to highlight SNP identity subgroups. (**A**) Using a strain from the earliest lineage identified during the epidemic, Guinea-1a (reference input: *Gueckedou_GIN_C05_KJ660348.2_2014)* a multi-genome *EvoDifference* print was generated using isolates from both early and late stages of the epidemic as listed. Database sequences are all Ebola/Zaire lineage 6 strains. Note, sublineage markers at bases 13856 and 15660. Only four sequences in the database had markers that differed from the input at positions 13856 or 15660 but not both

(data not shown). (**B**) Using an isolate from a late sublineage, designated Sierra Leone-Guinea-3 (input reference sequence *SLE_G3856.1_2014*), a multi-genome *EvoDifference* print was generated using isolates from both early and late stages of the epidemic as listed. The readout identifies a sublineage marker at base position 10218 as well as sublineage identity SNPs at 10248 and 10273 positions. The database entries are also grouped according to their country of origin. The reference sequence, with an A nucleotide at position 10218, is distinct from Guinea-1a lineage, which was generated early in the epidemic, with all other genomes having a G at position 10218. All isolates with an A at position 10218 are from Sierra Leone (total of 65 in the database) or Guinea (total of 19 in the database) while the Liberian strains have a G at position 10218 indicating their origin early during the course of the epidemic. Many sublineage-A sequences are highly diverged from the input reference sequence yet share with the input an A at position 10218 (total of 30 in the database). isolates with a G at position 10218 include 13 from Sierra Leone and 39 from Guinea (including 9 with a C at position 10248).
