# Supplementary material for: Flavivirus and Filovirus EvoPrinters: New alignment tools for the comparative analysis of viral evolution
Source: PLoS Negl Trop Dis. 2017 Jun 16;11(6):e0005673. doi: 10.1371/journal.pntd.0005673 (PMC5489223; doi:10.1371/journal.pntd.0005673)
Supplement: S8 Fig — One-on-one alignments of related Marburg strains highlight clusters of T/U->C base changes within the VP35 and VP40 ORFs. (A1) The Marburg_lin3_Ang_LakeVictoria_1381_DQ447654.1_2005 reference sequence from bases 2550 to 3300 including a portion of intragenic region between NP and VP35 and the start of the VP35 open reading frame (marked by the green carat) aligned with Marburg_lin3_Ang_KM261523.1_2005. The four expanded sequence lines reveals a cluster of 23 T/U -> C base substitutions extending 289 bases, 19 of which are in the intragenic region between NP and VP35 coding regions; four additional substitutions are found within the VP35 ORF. (A2 and A3) Of these four substitutions, two resulted in nonsynonymous amino acid changes, illustrated using the EvoPrinter translation utility. (B1) The Marburg_lin9_Kenya_LakeVictoria_Ravn_R1_EU500827.1_1987 reference sequence from bases 2776 to 3375 aligned with Marburg_lin9_Kenya EU500827.1_1987. Two sequence lines of the VP-35 coding sequence, that exhibit base substitutions (red highlight), have been expanded to reveal the substitution (T to C) in the Kenya isolate. All substitutions occurred in the open reading frame (the methionine-encoding ATG start site is marked with the green carat). (B2 and B3) As a result of the A to I editing, five nonsynonymous changes occurred in the encoded amino acids. (C1) The Marburg_lin9_DRG_DQ447652.1_1999 reference sequence from bases 4376 to 4875 of the region of the VP40 coding sequence is aligned to the orthologous region of Marburg_lin9_Kenya_EU500827.1_1987. Three sequence lines that exhibit base substitutions (red highlight) have been expanded to reveal the substitution (T->C) in the Kenya isolate. All but the first occurred in the open reading frame (the methionine-encoding ATG start site is marked with the green carat). (C2 and C3) As a result of the (T->C) substitutions nonsynonymous changes occurred in five encoded amino acids. (D1) The Marburg_lin9_Kenya_LakeVictoria_Ravn_R1_EU [file pntd.0005673.s008.pdf]

A1

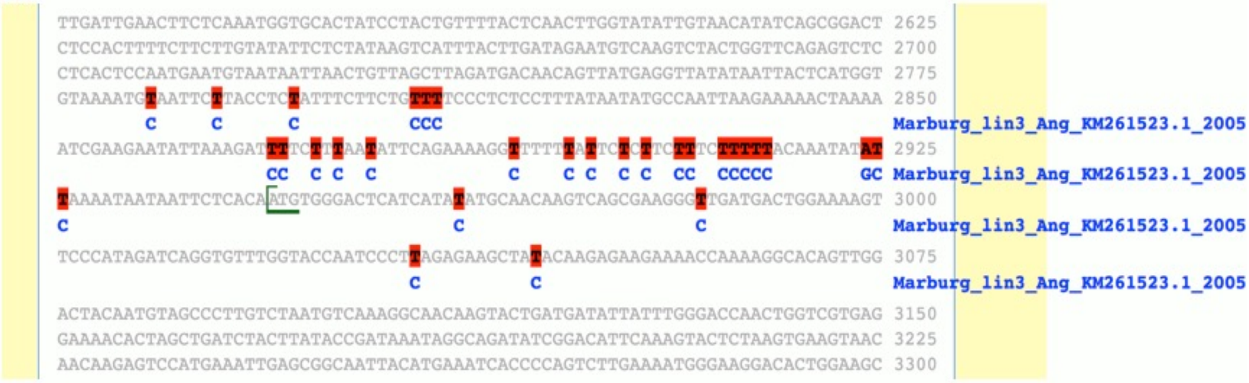
TTGATTGAACTTCTCAAATGGTGCACTATCCTACTGTTTTACTCAACTTGGTATATTGTAACATATCAGCGGACT 2625
CTCCACTTTTCTTCTTGTATATTCTCTATAAGTCATTTACTTGATAGAATGTCAAGTCTACTGGTTCAGAGTCTC 2700
CTCACTCCAATGAATGTAATAATTAACTGTTAGCTTAGATGACAACAGTTATGAGGTTATATAATTACTCATGGT 2775
GTAAAATGTAATTCTTACCTCTATTTCTTCTGTTTTCCCTCTCCTTTATAATATGCCAATTAAGAAAAACTAAAA 2850
Marburg_lin3_Ang_KM261523.1_2005
ATCGAAGAATATTAAAGATTTTCTTTAATATTCAGAAAAGGTTTTTTATTCTCTTCTTTCTTTTTACAAATATAT 2925
Marburg_lin3_Ang_KM261523.1_2005
TAAAATAATAATTCTCACAATGTGGGACTCATCATATATGCAACAAGTCAGCGAAGGGTTGATGACTGGAAAAGT 3000
Marburg_lin3_Ang_KM261523.1_2005
TCCCATAGATCAGGTGTTTGGTACCAATCCCTTAGAGAAGCTATACAAGAGAAGAAAACCAAAAGGCACAGTTGG 3075
Marburg_lin3_Ang_KM261523.1_2005
ACTACAATGTAGCCCTTGTCTAATGTCAAAGGCAACAAGTACTGATGATATTATTTGGGACCAACTGGTCGTGAG 3150
GAAAACACTAGCTGATCTACTTAATACCGATAAATAGGCAGATATCGGACATTCAAAGTACTCTAAGTGAAGTAAC 3225
AACAAGAGTCCATGAAATTGAGCGGCAATTACATGAAATCACCCCAGTCTTGAAAATGGGAAGGACACTGGAAGC 3300

A2

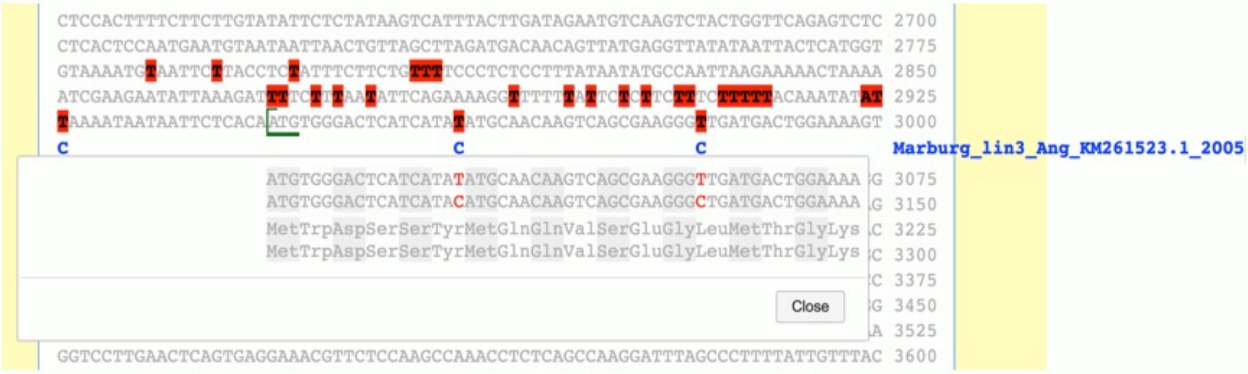
Marburg_lin3_Ang_KM261523.1_2005
ATGTGGGACTCATCATATATGCAACAAGTCAGCGAAGGGTTGATGACTGGAAAA
ATGTGGGACTCATCATACATGCAACAAGTCAGCGAAGGGCTGATGACTGGAAAA
MetTrpAspSerSerTyrMetGlnGlnValSerGluGlyLeuMetThrGlyLys
MetTrpAspSerSerTyrMetGlnGlnValSerGluGlyLeuMetThrGlyLys
Close
GGTCCTTGAACTCAGTGAGGAAACGTTCTCCAAGCCAAACCTCTCAGCCAAGGATTTAGCCCTTTTATTGTTTAC 3600

A3

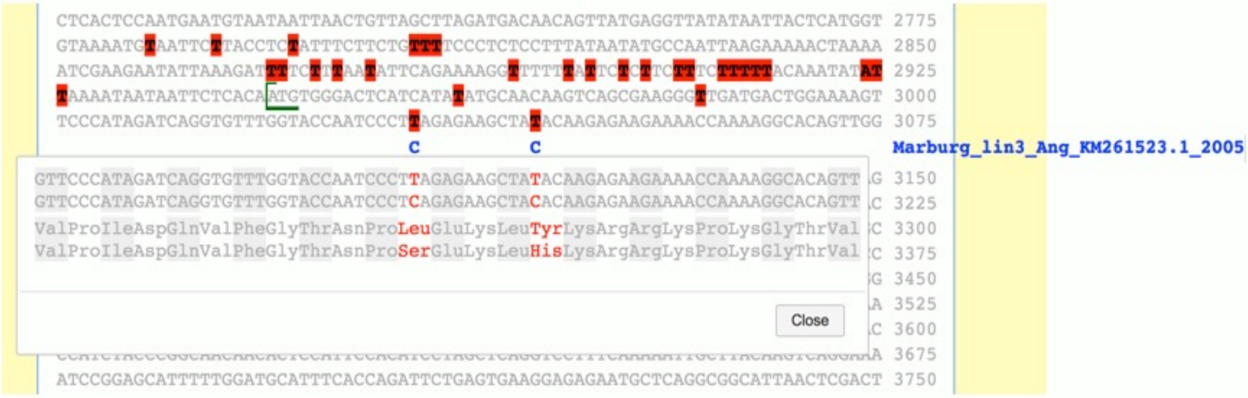
Marburg_lin3_Ang_KM261523.1_2005
GTTCCCATAGATCAGGTGTTTGGTACCAATCCCTTAGAGAAGCTATACAAGAGAAGAAAACCAAAAGGCACAGTT
GTTCCCATAGATCAGGTGTTTGGTACCAATCCCTCAGAGAAGCTACACAAGAGAAGAAAACCAAAAGGCACAGTT
ValProIleAspGlnValPheGlyThrAsnProLeuGluLysLeuTyrLysArgArgLysProLysGlyThrVal
ValProIleAspGlnValPheGlyThrAsnProSerGluLysLeuHisLysArgArgLysProLysGlyThrVal
Close
ATCCGGAGCATTTTTGGATGCATTTCACCAGATTCTGAGTGAAGGAGAGAATGCTCAGGCGGCATTAACTCGACT 3750

B1

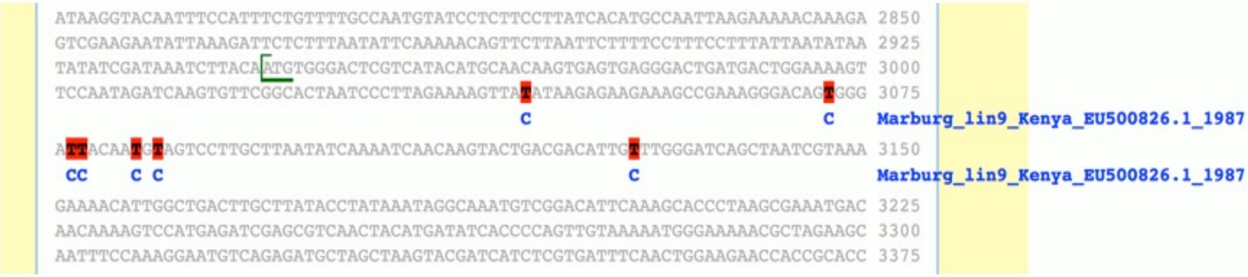
Marburg_lin9_Kenya_EU500826.1_1987
Marburg_lin9_Kenya_EU500826.1_1987

B2

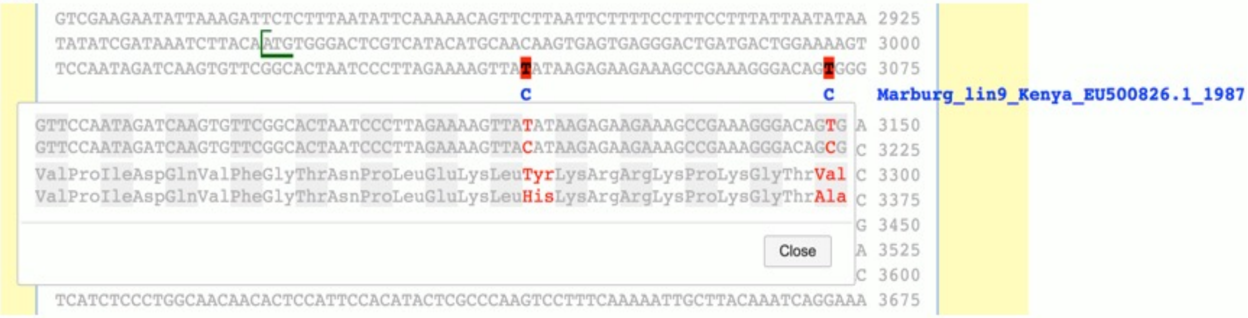
Marburg_lin9_Kenya_EU500826.1_1987
ValProIleAspGlnValPheGlyThrAsnProLeuGluLysLeuTyrLysArgArgLysProLysGlyThrVal
ValProIleAspGlnValPheGlyThrAsnProLeuGluLysLeuHisLysArgArgLysProLysGlyThrAla
Close

B3

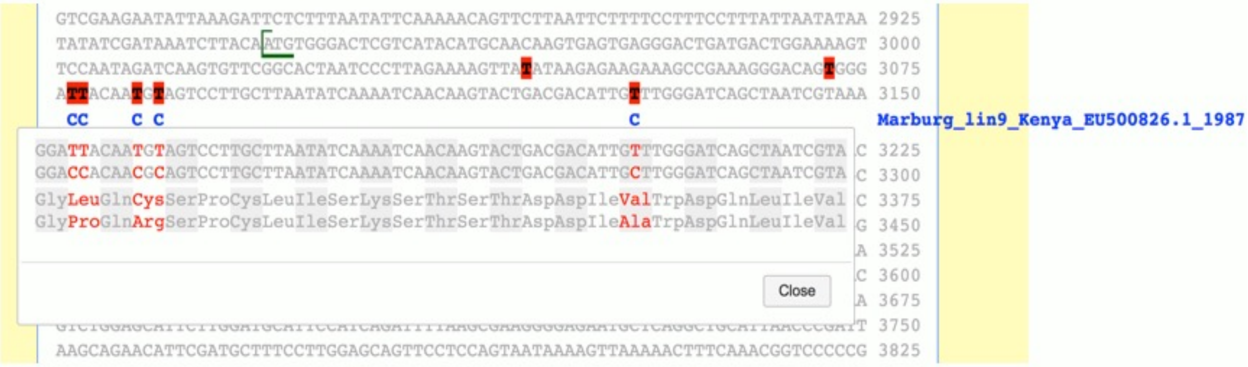
Marburg_lin9_Kenya_EU500826.1_1987
GlyLeuGlnCysSerProCysLeuIleSerLysSerThrSerThrAspAspIleValTrpAspGlnLeuIleVal
GlyProGlnArgSerProCysLeuIleSerLysSerThrSerThrAspAspIleAlaTrpAspGlnLeuIleVal
Close

C1

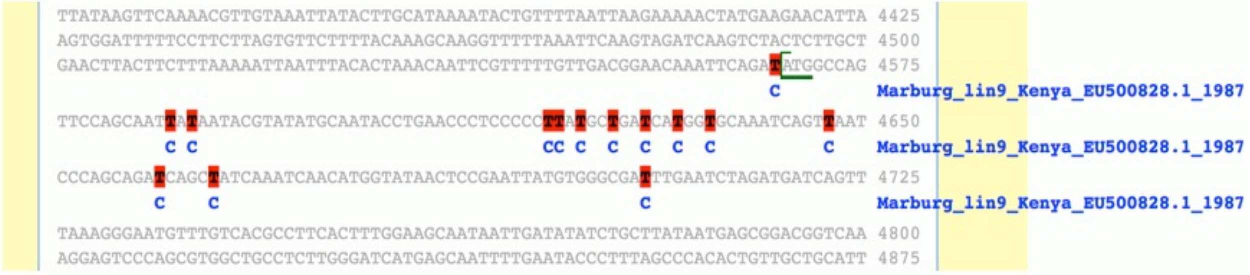
Marburg_lin9_Kenya_EU500828.1_1987
Marburg_lin9_Kenya_EU500828.1_1987
Marburg_lin9_Kenya_EU500828.1_1987

C2

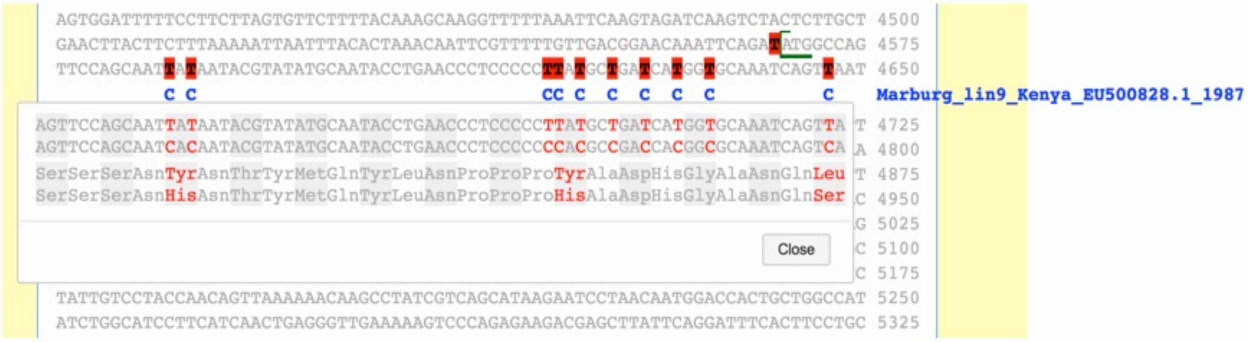
Marburg_lin9_Kenya_EU500828.1_1987
SerSerSerAsnTyrAsnThrTyrMetGlnTyrLeuAsnProProProTyrAlaAspHisGlyAlaAsnGlnLeu
SerSerSerAsnHisAsnThrTyrMetGlnTyrLeuAsnProProProHisAlaAspHisGlyAlaAsnGlnSer
Close

C3

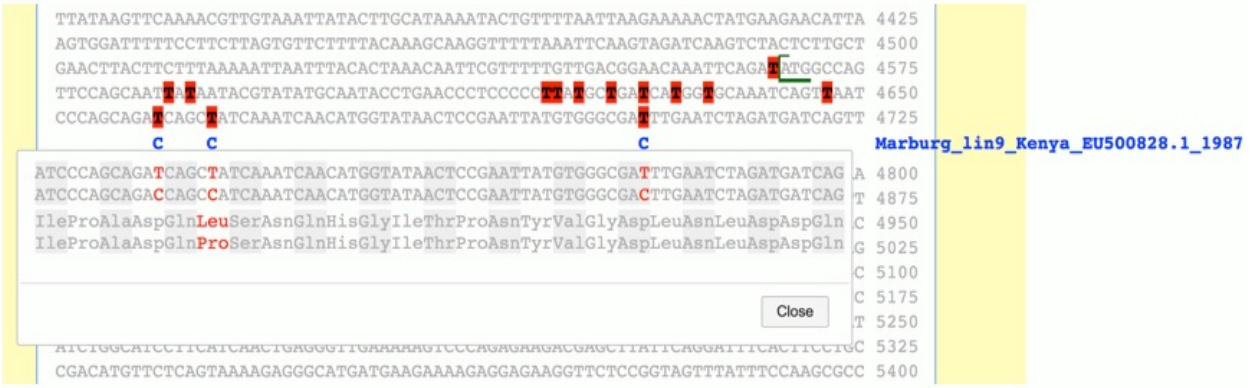
Marburg_lin9_Kenya_EU500828.1_1987
IleProAlaAspGlnLeuSerAsnGlnHisGlyIleThrProAsnTyrValGlyAspLeuAsnLeuAspAspGln
IleProAlaAspGlnProSerAsnGlnHisGlyIleThrProAsnTyrValGlyAspLeuAsnLeuAspAspGln
Close

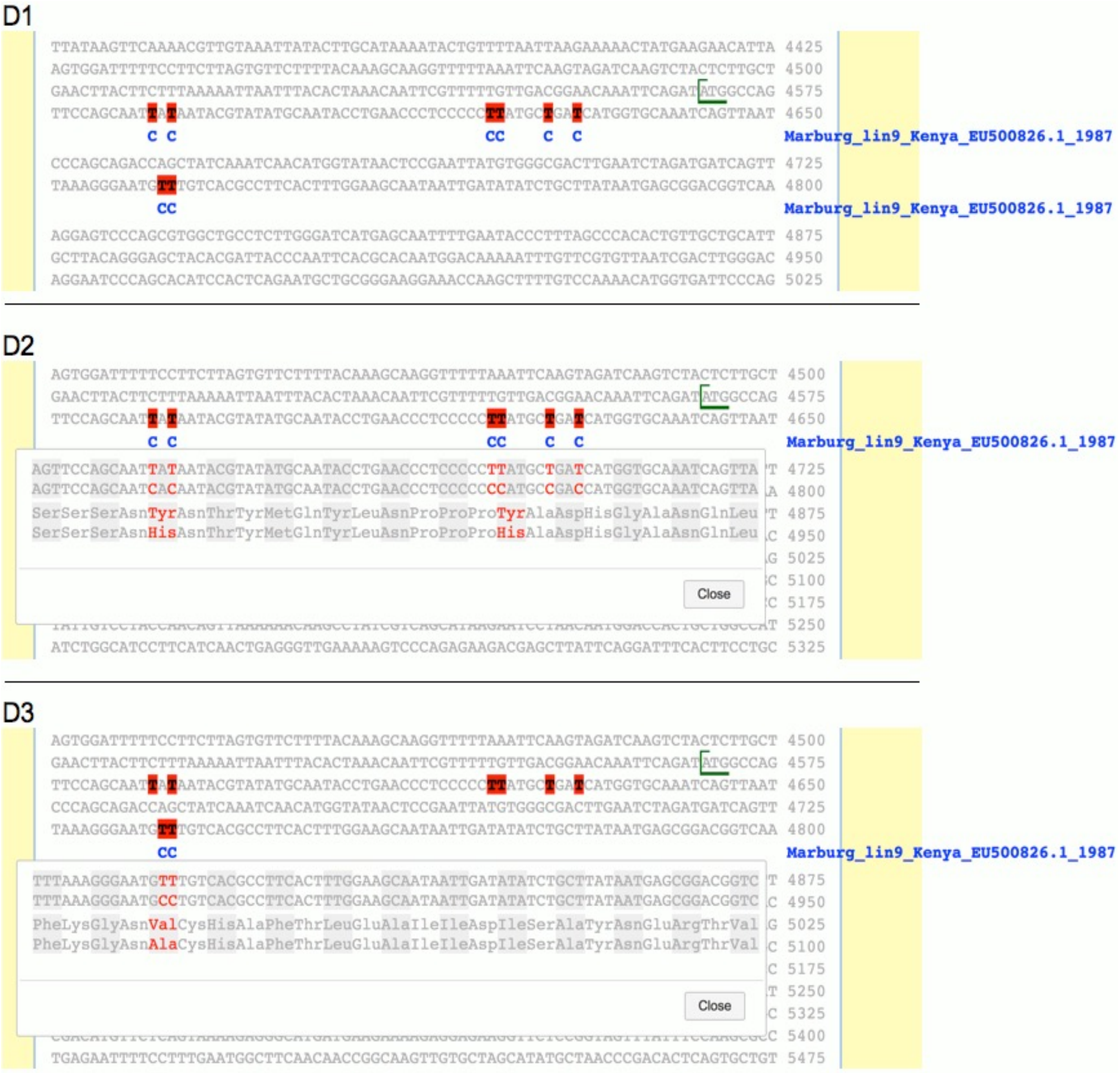

S8_fig.pdf A-to-I host cell hyper-editing in Marburg virus open reading frames detected using *EvoDifference* prints

One-on-one alignments of related Marburg strains highlight clusters of T/U->C base changes within the VP35 and VP40 ORFs. **(A1)** The *Marburg_lin3_Ang_LakeVictoria_1381_DQ447654.1_2005* reference sequence from bases 2550 to 3300 including a portion of intragenic region between NP and VP35 and the start of the VP35 open reading frame (marked by the green carat) aligned with *Marburg_lin3_Ang_KM261523.1_2005*. The four expanded sequence lines reveals a cluster of 23 T/U -> C base substitutions extending 289 bases, 19 of

which are in the intragenic region between NP and VP35 coding regions; four additional substitutions are found within the VP35 ORF. (**A2** and **A3**) Of these four substitutions, two resulted in nonsynonymous amino acid changes, illustrated using the *EvoPrinter* translation utility. (**B1**) The *Marburg_lin9_Kenya_LakeVictoria_Ravn_R1_EU500827.1_1987* reference sequence from bases 2776 to 3375 aligned with *Marburg_lin9_Kenya EU500827.1_1987*. Two sequence lines of the VP-35 coding sequence, that exhibit base substitutions (red highlight), have been expanded to reveal the substitution (T/U -> C) in the Kenya isolate. All substitutions occurred in the open reading frame (the methionine-encoding ATG start site is marked with the green carat). (**B2** and **B3**) As a result of the A-to-I editing, five nonsynonymous changes occurred in the encoded amino acids. (**C1**) The *Marburg_lin9_DRG_DQ447652.1_1999* reference sequence from bases 4376 to 4875 of the region of the VP40 coding sequence is aligned to the orthologous region of *Marburg_lin9_Kenya_EU500827.1_1987*. Three sequence lines that exhibit base substitutions (red highlight) have been expanded to reveal the substitution (T->C) in the Kenya isolate. All but the first occurred in the open reading frame (the methionine-encoding ATG start site is marked with the green carat). (**C2** and **C3**) As a result of the (T->C) substitutions nonsynonymous changes occurred in five encoded amino acids. (**D1**) The Marburg_lin9_Kenya_LakeVictoria_Ravn_R1_EU500827.1_1987 reference sequence from bases 4365 to 5025 aligned with Marburg_lin9_Kenya_EU500826.1_1987 to reveal T/U -> C base substitutions in a VP40 coding sequence. Two sequence lines that exhibit the base substitutions (red highlight) have been expanded to reveal the substitutions in the Marburg_lin9_Kenya EU500827.1_1987 isolate. All T to C substitutions in this cluster occurred in the open reading frame (ATG start site is marked with the green carat). (**D2** and **D3**) As a result of the A-to-I editing, three nonsynonymous changes were detected that result in amino acid changes.
